# Muscle Phenotype, Proteolysis, and Atrophy Signaling During Reloading in Mice: Effects of Curcumin on the Gastrocnemius

**DOI:** 10.3390/nu12020388

**Published:** 2020-01-31

**Authors:** Laura Mañas-García, Nuria Bargalló, Joaquim Gea, Esther Barreiro

**Affiliations:** 1 Pulmonology Department-Muscle Wasting and Cachexia in Chronic Respiratory Diseases and Lung Cancer Research Group, IMIM-Hospital del Mar, Parc de Salut Mar, Barcelona Biomedical Research Park (PRBB), 08003 Barcelona, Spain; laura.manas@upf.edu (L.M.-G.); nrbargallo@gmail.com (N.B.); jgea@parcdesalutmar.cat (J.G.); 2 Health and Experimental Sciences Department (CEXS), Universitat Pompeu Fabra (UPF), Barcelona Biomedical Research Park (PRBB), 08003 Barcelona, Spain; 3 Centro de Investigación en Red de Enfermedades Respiratorias (CIBERES), Instituto de Salud Carlos III (ISCIII), 08003 Barcelona, Spain

**Keywords:** disuse muscle atrophy, muscle reloading, limb muscle, sirtuin-1, curcumin, proteolysis and apoptosis, acetylation of atrophy signaling pathways

## Abstract

We hypothesized that curcumin may mitigate muscle protein degradation and loss through attenuation of proteolytic activity in limb muscles of mice exposed to reloading (7dR) following immobilization (7dI). In gastrocnemius of mice (female C57BL/6J, 10 weeks) exposed to recovery following a seven-day period of hindlimb immobilization with/without curcumin treatment, markers of muscle proteolysis (systemic troponin-I), atrophy signaling pathways and histone deacetylases, protein synthesis, and muscle phenotypic characteristics and function were analyzed. In gastrocnemius of reloading mice compared to unloaded, muscle function, structure, sirtuin-1, and protein synthesis improved, while proteolytic and signaling markers (FoxO1/3) declined. In gastrocnemius of unloaded and reloaded mice treated with curcumin, proteolytic and signaling markers (NF-kB p50) decreased and sirtuin-1 activity and hybrid fibers size increased (reloaded muscle), while no significant improvement was seen in muscle function. Treatment with curcumin elicited a rise in sirtuin-1 activity, while attenuating proteolysis in gastrocnemius of mice during reloading following a period of unloading. Curcumin attenuated muscle proteolysis probably via activation of histone deacetylase sirtuin-1, which also led to decreased levels of atrophy signaling pathways. These findings offer an avenue of research in the design of therapeutic strategies in clinical settings of patients exposed to periods of disuse muscle atrophy.

## 1. Introduction

In chronic disease and cancer, disuse muscle atrophy is common in the affected patients as a result of physical inactivity [1,2,3,4,5]. Patients with chronic diseases are also exposed to periods of prolonged bed rest due to acute exacerbations, which further contribute to worsen muscle atrophy in those individuals. Moreover, muscle atrophy is also common during critical illness that further impairs muscle mass in the patients. In the elderly, bone fractures and surgical interventions are frequent, which altogether deteriorates muscle mass and performance in the patients. Thus, disuse muscle atrophy imposes a tremendous burden on the health-care systems as the recovery of muscle mass and function can only be attained following strict long-term rehabilitation programs. 

Importantly, muscle wasting and loss of function were demonstrated to have prognosis value in terms of morbidity and mortality in patients with chronic diseases [6,7,8,9,10,11] and in cancer [12,13,14,15]. Muscle atrophy and dysfunction are predictors of survival regardless of the status of the underlying condition [2,3,4,5]. Furthermore, disability following acute exacerbations of chronic diseases and/or critical illness is very common as the full recovery of muscle mass and function is barely achieved, especially in the elderly and physically inactive patients. 

In the multifactorial etiology of muscle atrophy in patients and animal models, factors inherent to the disease and biological mechanisms are altered leading to the loss of muscle mass and function. Our group and others have published extensively in the elucidation of the mechanisms that underlie the process of muscle wasting and impaired function. As such, in models of disuse muscle atrophy and wasting, a rise in the levels of markers of proteolysis, autophagy, apoptosis, oxidative stress, and epigenetic modifications have been shown in muscles of both patients [2,6,7,8,9,10,11] and animals [12,13,14,16,17,18,19,20,21,22]. Additionally, alterations in the structure of the myofibers along with a reduction in their size have also been demonstrated in muscles following periods of inactivity [12,13,14,16,17,18,19,20,21,22]. 

The study of the kinetics of the pathophysiological and biological events whereby the loss of muscle mass takes place following periods of disuse is important [23,24,25]. In this regard, the sequence of the expression of markers of proteolysis, apoptosis, autophagy, signaling, of structural alterations and fiber type switches, and that of impaired function have already been described in previous investigations [23,24,25]. 

Post-translational modifications of transcription factors that signal proteolytic activation in muscles were described in several models of muscle atrophy [24,25,26]. The kinetics of acetylation status of the transcription factors fork-head box O (FoxO)1 and FoxO3 were explored in the gastrocnemius muscle of mice exposed to several periods of immobilization followed by periods of recovery [24,25]. Furthermore, inactivity of the limb muscle induced a decline in levels of the histone deacetylase sirtuin-1, whereas muscle reloading led to an increase in protein levels of this enzyme [24,25]. Sirtuin-1 has been shown to play a key role in the protection against myocardial infarction [27] and in the prevention of the senescence of the vasculature in cells [28]. Whether an increase in sirtuin-1 levels may attenuate protein degradation during muscle unloading remains to be fully evaluated.

Curcumin, a polyphenolic compound derived from turmeric, was shown to exert beneficial effects in several models. As such, curcumin protected against myocardial infarction-induced fibrosis via sirtuin-1 activation in mice and cells [27]. Curcumin also elicited a rise in sirtuin-1 levels of senescent smooth muscle and endothelial cells [28]. Whether curcumin therapy may induce favorable effects on muscle protein breakdown and structure in disuse muscle atrophy and reloading has not yet been identified. 

Thus, we hypothesize that curcumin may mitigate muscle protein degradation and mass loss through attenuation of the action of proteolysis, atrophy signaling pathways, apoptosis, structural alterations, and muscle performance in limb muscles of mice exposed to reloading following a period of unloading. Accordingly, the study objectives were that in the gastrocnemius of mice exposed to recovery for seven days following a seven-day period of hindlimb immobilization with and without treatment with curcumin, several molecular events involved in muscle mass maintenance were explored: 1) markers of proteolysis including systemic levels of troponin-I, 2) atrophy signaling pathways and histone deacetylases, 3) protein synthesis, and 4) muscle structure and function. The experimental model used in the present study has been previously well-validated [23,24,25]. 

## 2. Methods 

### 2.1. Animal Experiments

In this study, C57BL/6J mice (10 weeks old, weight ~20 g, females) were purchased from Harlan *Interfauna Ibérica SL* (Barcelona, Spain). All the animals were maintained under a pathogen-free environment in the animal house facility at the Barcelona Biomedical Research Park (PRBB), with a 12:12 h light:dark cycle. 

**Figure 1:**
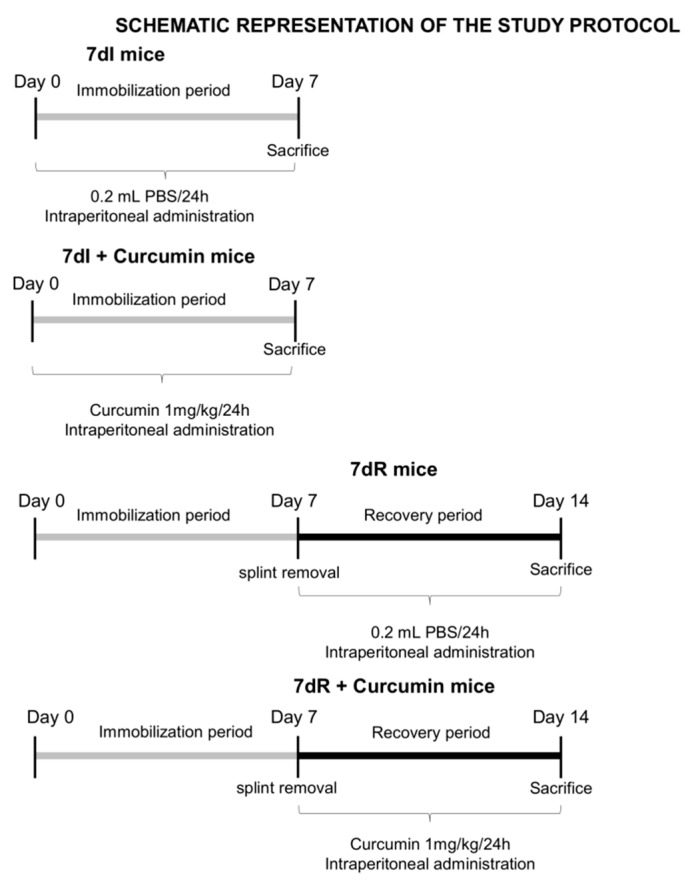
Schematic representation of the study protocol and groups of animals as well as of the different therapeutic approaches.

The study protocol is illustrated in Figure 1. Unilateral hindlimb immobilization was applied to rodents as previously reported with the aim to mimic disuse muscle atrophy [23,24,25]. Essentially, clippers were used to shave the left hindlimb, which was subsequently protected with surgical tape. Microcentrifuge tubes of 1.5 mL (0.6 g) were used in the study. The cover and bottom lids were removed for the hindlimb to be introduced. The foot of the mice was kept in a plantar-flexed position in order to elicite the greatest degree of muscle atrophy [24,25]. The mice were able to move freely in the cages even those wearing the plastic splint. The current experimental model has already been well validated as shown in previous investigations [24,25]. The degree of atrophy attained in the gastrocnemius muscle has consistently ranged between 18% to 25% for both slow- and fast-twitch muscle fibers. On this basis, the level of muscle atrophy was confirmed again (5 mice) to be 25% in the gastrocnemius muscle of immobilized mice in the current study. However, for the sake of clarity and conciseness those animals have not been included in the present investigation. 

As such, the following groups of mice were investigated (n = 10/group, Figure 1): 1) 7 days immobilized mice (7dI, left hindlimb immobilized for seven consecutive days), 2) 7 days recovery mice (7dR, left hindlimb immobilized for seven consecutive days, when the plastic splint was removed and the animals were moving free in their cages, to assess muscle recovery), 3) 7dI mice treated with curcumin (7dI+Curcumin, intraperitoneal administration, 1mg/kg weight/24 h) [29] from day 0 to day 7, and 4) 7dR mice treated with curcumin (7dR+Curcumin, intraperitoneal administration, 1mg/kg weight/24 h) from day 7 to day 14 (Figure 1). The protocol described by Vazeille et al [29] was followed to establish the dose and methodologies on how to prepare curcumin to be administered to the mice. Briefly, 1mg/kg weight/24 h of the compound curcumin was administered intraperitoneally to the mice [29]. Moreover, the half-life of circulating curcumin was previously established to go from 15 to 60 min in animal models and patients [30,31]. Additional experiments of all the study groups of mice were carried out in order to quantify the status of protein synthesis in all the muscles. As such, mice were injected puromycin (intraperitoneally 0.04 μmol/g body weight) 30 min prior to sacrifice. Samples from these animals were also collected (see below) [32].

### 2.2. Ethics

Experiments involving the use of animals were all carried out in the animal facilities at our center (PRBB). A controlled investigation was designed following the ethical regulations on animal experimentation in Europe (European Community Directive 2010/63/EU), Spain (Spanish Legislation, *Real Decreto* 53/2013, BOE 34/11370–11421), and the European Convention for the Protection of Vertebrate Animals Used for Experimental and Other Scientific Purposes (1986). The Animal Research Committee at PRBB approved the animal studies (Animal Welfare Department in Catalonia, Spain, EBP-13-1485).

### 2.3. Studies in Mice: In Vivo Measurements

The parameters body weight and food intake were obtained at every time-point for all the mice. In both immobilization and recovery phases of the study, food and water were administered ad libitum. The parameter limb strength was determined using a grip strength meter (Bioseb, Vitrolles Cedex, France) on the following time-points: day 0, at the end of immobilization period, and at the end of the recovery phase as previously reported [18,19,24,26,33]. In this study, the four limbs were used to measure grip strength in all study groups. Limb strength gain was estimated as follows: (grip strength at the end of the study period–grip strength on day 0)/grip strength on day 0 x 100 [24,25]. 

### 2.4. Mouse Sacrifice and Collection of the Samples

Following the immobilization or recovery periods (7 or 14 days), all the animals were sacrificed in the animal facilities. Sodium pentobarbital (60 mg/Kg, 0.1 mL) was administered five minutes prior to starting the sacrifice experiments. The pedal and blink reflexes were checked to confirm complete anesthesia. The gastrocnemius was dissected and collected from all the mice during sacrifice. Liquid nitrogen was used to snap-freeze the muscle specimens, which were further stored in the −80 °C freezers. Molecular analyses were conducted at a later stage, once the samples from all the groups had been obtained. Moreover, for histological analyses two additional fragments of the muscle specimens were paraffin-embedded for apoptosis assay, and in optimum cutting temperature (OCT) for fiber-typing and morphometrical analyses.

### 2.5. Muscle Biology

**Figure 2:**
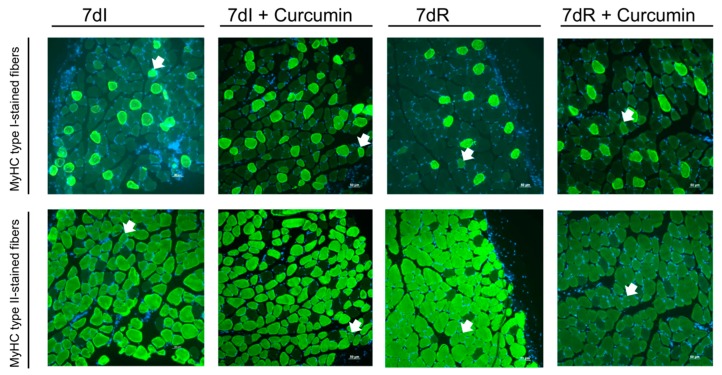
Representative examples of the gastrocnemius muscle in animals of the all study groups of mice. Myofibers were stained in green, type I in the top panel and type II in the bottom panel. Hybrid fibers (arrows) are seen in both panels. Definition of abbreviations: MyHC myosin heavy chain; I, immobilization; R, recovery.

*Muscle fiber typing and morphometrical analyses.* Muscle samples were fixed in 4% paraformaldehyde solution, pH 6.9 (EMD Millipore corporation, Billerica, MA, USA). They were subsequently embedded in increasing concentrations of sucrose. Additionally, the samples were also embedded in tissue-tek OCT compound (Sakura Finetek, Torrance, CA, USA). Thereafter they were snap-frozen in 2-methyl-butane immersed in liquid nitrogen as previously reported [34]. A cryostat-microtome (Leica CM3050S, Leica Biosystems, Wetzlar, Germany) was used to cut ten-µm frozen sections (−20 ℃), to be thereafter mounted on glass slides. Immunofluorescence procedures with specific antibodies (anti-myosin heavy chain (MyHC) I and anti-MyHC II, respectively) were used to determine slow- and fast-twitch muscle fibers in each preparation (Figure 2 and Figure S1). The following protocol was used: muscle cross-sections were air-dried for thirty minutes and were rinsed with phosphate-buffered saline (PBS) for another fifteen minutes. Following rising, the sections were stored in cold methanol for six more minutes. Immediately afterwards, a 20 minute period of boiling in a pressure cooker [10 mM citrate buffer (pH 6.0)] buffer followed. Samples were then cooled down to room temperature for two hours. The sections were then incubated with mouse IgG blocking reagent (MOM, Vector Laboratories, Burlingame, CA, USA) for one hour, as well as in blocking solution (3% Bovine serum albumin (BSA), 10% goat serum and 0.5% triton in PBS) for another hour. Later, they were incubated overnight with the mouse monoclonal anti-MyHC I antibody (ab11083, Abcam), and anti-MyHC II antibody (ab51263, Abcam) prepared in blocking solution at 4℃. Following incubation with the primary antibody and after rising with PBS, the sections were incubated with the corresponding secondary antibody and with 4’, 6-diamino-2-fenilindol (DAPI, which specifically stained DNA allowing identification of all nuclei) for one hour at room temperature: anti-mouse IgG1 Alexa Fluor 488 antibody, which was also prepared in the blocking solution. Additionally, negative control experiments were carried out by omission of the primary antibody and incubation of the muscle samples only with secondary antibody, was also performed to confirm the specificity of each antibody (Figure S1). 

Finally, the sections were mounted using 70% glycerol in 30% PBS. In two consecutives muscle cross-sections, muscle fibers positively stained with the anti-MyHC type I antibody or anti-MyHC type II were those that appeared as fluorescein isothiocyanate (FITC)- stained in green. The following parameters were determined in each muscle cross-section: mean least diameter, cross-sectional area, and the proportions of type I and type II using a fluorescence microscope (x20 objective, Nikon Eclipse Ni, Nikon, Tokyo, Japan) coupled with and image-digitizing camera (Zyla 4.2 sCMOS camera, Andor, Belfast, UK) and the Image J software (National Institute of Health, available at http://rsb.info.nih.gov/ij/). At least 100 fibers were measured and counted, individually, in each muscle cross-section of all the animals. Fibers that were stained simultaneously for both anti-type I and anti-type II primary antibodies were identified as the hybrid fibers. They were all counted in the histological preparations of all the study groups (Figure 2 and Figure S1).

*Terminal deoxynucleotidyl transferase-mediated uridine 5’-triphosphate (UTP) nick-end labeling (TUNEL) assay.* The number of apoptotic nuclei were identified using the TUNEL assay (ApopTag® Peroxidase In Situ Apoptosis Detection Kit, Meck-Millipore, Darmstadt, Germany) in paraffin-embedded muscle sections of all the study animals as previously reported [18,26,33,35]. The muscle sections were fixed and permeabilized to be then incubated with the TUNEL reaction mixture (terminal deoxynucleotidyl transferase (TdT) and fluorescein-dUTP, Figure S2). TdT catalyzed the addition of fluorescein-dUTP at free 3’-OH groups in single- and double-stranded DNA during the incubation phase. The label incorporated at the damaged sites of the DNA was marked by an anti-fluorescein antibody conjugated with the reporter enzyme peroxidase following several washes. The peroxidase kept in the immune complex was visualized by a substrate reaction. Additionally, omission of the reaction mixture was also performed in several experiments as the negative control. 

**Figure 3:**
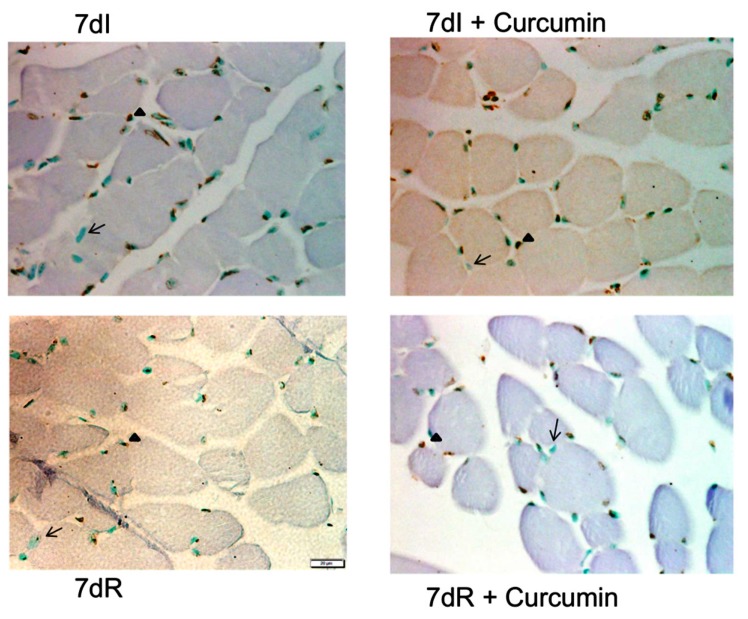
Representative examples of positively-stained nuclei (arrowheads) and negatively-stained nuclei (arrows) for the TUNEL assay in the gastrocnemius muscles of all the study groups of mice. Definition of abbreviations: I, immobilization; R, recovery.

In the histological preparations, the positively-stained nuclei appeared in brown, while negative nuclei were non-stained (blue color, methyl-green counterstaining, (Figure 3 and Figure S2). TUNEL positive nuclei were those clearly located within the muscle fiber boundary in each section. Moreover, localization of actual myofibers within the muscle sections was performed by identification of dystrophin protein with a specific antibody (d8168, clone MANDYS8, Sigma-Aldrich). Myonuclei were identified inside the dystrophin-limited boundaries in each preparation (Figure S2). 

The TUNEL-positive nuclei and the total number of nuclei were counted blind by two independent observers in each muscle sample that were expressed as follows: the percentage of the TUNEL-positive nuclei from the total number of counted nuclei, as also previously shown [14,18]. In each muscle section, at least 300 nuclei were counted. 

*Immunoblotting of 1D electrophoresis*. Immunoblotting was used to identify the target markers in the study. Previously published procedures by our group and others were followed [18,24,25]. Muscle specimens from the gastrocnemius were homogenized in a specific buffer as previously reported [18,24,25]: 50 mM 4-(2-hydroxyethyl)-1-piperazineethanesulfonic acid (HEPES), 150 mM NaCl, 100 mM NaF, 10 mM Na pyrophosphate, 5 mM ethylenediaminetetraacetic acid (EDTA), 0.5% Triton-X, 2 micrograms/mL leupeptin, 100 micrograms/mL phenylmethanesulfonyl fluoride (PMSF), 2 micrograms/mL aprotinin and 10 micrograms/mL pepstatin A. Additionally, levels of actin and MyHC proteins were identified using a specific homogenization protocol as previously shown [25].

All the experiments were carried out at 4 ℃. Bradford methodologies were used to determine protein concentration in triplicates for each sample. BSA was used as the standard (Bio-Rad protein reagent, Bio-Rad Inc., Hercules, CA, USA). Equal amounts of total protein (ranging from 5 to 200 micrograms, according to antigen and antibody) from crude muscle homogenates were loaded for all the gels, as well as identical sample volumes/lanes. Muscle specimens were always run together for the four study groups (38 study samples and two lanes for the molecular markers) and were kept in the same order in the gels of mini-cell boxes for the sake of comparisons. Four fresh 10-well mini-gels were run in the mini-cell boxes for each of the antigens. The samples were always transferred and detected together simultaneously for all the antigens analyzed in the investigation (see raw immunoblots in the online supplement, Figures S3–S26). Experiments were confirmed at least twice for all the antigens analyzed in the investigation. 

Muscle proteins were separated by electrophoresis, were subsequently transferred to polyvinylidene difluoride (PVDF) membranes, and were then blocked with BSA to be incubated with selective primary antibodies overnight. Protein levels of the following markers and pathways were analyzed in the study muscles: total acetylated proteins, histone deacetylases (HDACs), signaling pathways including phosphorylation, structural proteins, and downstream targets. The following specific primary antibodies: total acetylated proteins (anti-acetyl-lysine antibody, Santa Cruz Biotechnology, Santa Cruz, CA, USA), NAD-dependent protein deacetylase sirtuin-1 (anti-sirtuin-1 antibody, ProteinTech Group Inc., Rosemont, Illinois, USA), nuclear factor kappa-light-chain-enhancer of activated B cells (NF-κB) p50 (anti-p50 antibody, Santa Cruz Biotechnology), FoxO1 (anti-FoxO1 antibody, Merck-Millipore, Darmstadt, Germany), phospho-FoxO1 (Ser256) (anti-phospho-FoxO1 (Ser256) antibody, Santa Cruz Biotechnology), FoxO3 (anti-FoxO3 antibody, Origene, Herford, Germany), phospho-FoxO3 (Ser253) (anti-phospho-FoxO3 (Ser253) antibody, Merck-Millipore), peroxisome proliferator-activated receptor gamma coactivator 1-alpha (PGC-1α) (anti-PGC-1α antibody, Santa Cruz Biotechnology), MyHC (anti-MyHC antibody, clone A4.1025, Upstate-Millipore, Temecula, CA, USA), α-actin (anti-alpha-sarcomeric actin antibody, clone 5C5, Sigma-Aldrich, St. Louis, MO, USA) total ubiquitinated proteins (anti-ubiquitinated proteins antibody, Boston Biochem, Cambridge, MA, USA), 20S proteasome subunit C8 (anti-C8 antibody, Biomol, Plymouth Meeting, PA, USA), ubiquitin-ligase atrogin-1 (anti-atrogin-1 antibody, Acris), ubiquitin-ligase muscle ring finger (MuRF)-1 (anti-MuRF-1 antibody, Santa Cruz Biotechnology), puromycin (anti-puromycin antibody, clone 12D10, Merck-Millipore), Serine/Threonine Kinase 1 (Akt) (anti-Akt antibody, Cell Signaling Techonology, Leiden, The Netherlands) phospho-Akt (Ser473) (anti-phospho-Akt (Ser473), Cell Signaling Technology) HDAC3 (anti-HDAC3 antibody, Santa Cruz), HDAC4 (anti-HDAC4 antibody; Santa Cruz) and HDAC6 (anti-HDAC6 antibody; Abcam, Cambridge, UK) and glyceraldehyde-3-phosphate dehydrogenase (GAPDH, anti-GAPDH antibody, Santa Cruz Biotechnology). Horseradish peroxidase (HRP)-conjugated secondary antibodies and a chemiluminescence kit were used to detect the different antigens on the membranes. Samples from the different groups were always detected in the same picture under identical exposure times for each of the antigens. 

Omission of the primary antibody and incubation of the membranes only with secondary antibodies was used to confirm the specificity of the different antibodies. Acetylation levels of the transcription factors FoxO1, FoxO3, NF-kB, and PGC-1 alpha were detected as previously described [24,25]. The ratios of phosphorylated Akt, FoxO1, and FoxO3 to total levels of the corresponding transcription factors was calculated and are represented in graphs. 

Molecular Imager Chemidoc XRS System (Bio–Rad Laboratories, Hercules, CA, USA) and the software Quantity One version 4.6.5 (Bio–Rad Laboratories) were used to scan and analyze protein levels on the PVDF membranes. The software Image Lab version 2.0.1 (Bio-Rad Laboratories) was used to quantify the optical densities. For each of the study antigens, mean values of the different samples (lanes) were the final optical densities used in the statistical analyses. GAPDH was used to validate equal protein loading in all the immunoblots. Positive controls were used to assess the specific band in FoxO3 (FoxO3 HEK293T cell transient overexpression lysate, Origene), ubiquitin-ligase atrogin-1 (MAFBx 239T lysate, Santa Cruz Biotechnology), MuRF-1 (MuRF-1 239T lysate, Santa Cruz Biotechnology) and NF-κB p50 (NF-κB p50 293T lysate, Santa Cruz Biotechnology). 

*Stripping methodologies*. Primary and secondary antibodies were stripped off proteins following a 30 minute wash with a specific stripping solution [25 mM glycine, pH 2.0 and 1% sodium dodecyl sulfate (SDS)]. Immedately afterwards, two consecutive 10 minute washes of phosphate buffered saline with tween (PBST) were performed at room temperature. Subsequently, membranes were blocked with bovine BSA to be reincubated with primary and secondary antibodies of the target marker following the methodologies described above. 

*Protein catabolism.* The rate of production of free tyrosine was used to determine protein degradation in each muscle specimen [25,36,37,38]. Its accumulation reflects the net degradation of proteins, since muscles cannot synthesize or degrade this amino acid. All incubations were performed at 37 °C in a 95% air-5% CO_2_ mixture. Briefly, whole excised muscles were placed in individual tissue chambers containing 3 mL of TKH1 buffer (127.8 mM NaCl, 4.7 mM KCl, 2.4 mM MgSO4·7H2O, 1.2 mM KH2PO4, 2.5 mM CaCl_2_·2H_2_O, 20 mM HEPES, 170 µM L-leucine, 100 µM L-isoleucione, 200 µM L-valine and 0.5 M glucose) and were preincubated for 15 min. After the preincubation, the buffer was extracted and 3 mL of TKH2 (TKH1 supplemented with 500 mM cycloheximide) buffer were added to the same chamber, and samples were then incubated for 15 min. Thereafter, the buffer was extracted and replaced with 4 mL of fresh TKH2, and samples were then incubated for two hours. Immediately afterwards, the buffer was recovered and stored at −20 °C until the tyrosine release measurements were performed as described as follows: 1.4 mL of sample, blank (TKH2 buffer) or standards (tyrosine in blank from 0 to 2.5 µg/mL) were combined with 250 µL of 30% TCA in a centrifuge tube and centrifuged at 2500 rpm for 15 min, supernatant was recovered and placed on a new tube. Supernatant was combined with 300 µL of 1-nitroso-2-naphthol 0.1% in 95% ethanol and 300 µL of nitric acid mixture, and incubated for 30 min at 55 °C and then 15 min at room temperature. Four mL of ethylene dichloride were added to the tubes and were then shaken. The tubes were then centrifuged at 1500 rpm for 4 minutes. Four-hundred microliters of supernatant were transferred to a 96-well black microplate and measurements of tyrosine fluorescence was performed at 570 nm, resulting from its activation at 460 nm using a fluorometer (Infinite M200, TECAN, Männedorf, Switzerland). The results were expressed as nmol of tyrosine/mg of muscle/2 hours of incubation. 

*Skeletal muscle troponin-I levels in plasma.* In all the study groups of animals, skeletal muscle troponin-I levels were quantified in plasma samples: 7dI, 7dI+Curcumin, 7dR, 7dR+Curcumin, using a specific sandwich ELISA kit (Life Diagnostics Inc., West Chester, PA, USA) as previously described [25,39,40,41,42]. Initially, samples and reagents were equilibrated to room temperature. A standard curve was always run with each assay run. Standards (100 µL) were loaded and the protocol was followed as indicated by the manufacturer’s instructions. All reagents used in these experiments were part of the specific ELISA kit. For all the study samples equal volume (100 microL total volume) of diluted plasma (1:3 dilution) were always loaded in duplicates onto the pre-coated ELISA-plate wells. Samples were incubated with 100 microL HRP-secondary antibody on an orbital micro-plate shaker at 150 rpm and 25 °C for one hour. The wells were then washed six times with the wash solution and incubated with 100 microL tetramethylbenzidine (TMB) reagent on an orbital micro-plate shaker at 150 rpm and at 25 °C for 20 minutes. Finally, the enzyme reaction was stopped by adding 100 microL stop solution to the wells. A microplate reader was used to read the absorbance in each plasma sample at 450 nm (655 nm reference filter). Intra-assay coefficients of variation for the plasma skeletal muscle troponin-I levels ranged from 2% to 10%. As all the samples were analyzed on the same day, no inter-assay coefficients of variation could be calculated. 

*Sirtuin-1 activity.* Muscle homogenates were prepared from the frozen specimens using a specific buffer that contained the following reagents: 50 mM HEPES, 150 mM NaCl, 100 mM NaF, 10 mM Na pyrophosphate, 5 mM EDTA, 0.5% Triton-X, with no protease inhibitors. Homogenates were centrifuged in a 4 mL buffer containing 30% sucrose, 10 mM Tris HCl (pH 7.5), 10 mM NaCl, and 3 mM MgCl_2_ at 1300 *g* and at 4 °C for 10 min. The pellets were washed with cold 10 mM Tris-HCl (pH 7.5) and 10 mM NaCl, to be subsequently centrifuged at 1300 *g* and at 4 °C for 10 min. The resultant pellets, which contained the nuclei, were resuspended in 200 microL of extraction buffer containing 50 mM HEPES potassium hydroxide (HEPES KOH, pH 7.5), 420 mM NaCl, 0.5 mM EDTA, 0.1 mM EGTA, and 10% glycerol. The nuclei were subsequently sonicated in 15 s cycles. Afterwards, the sonicated nuclei remained on ice for 30 min. Following centrifugation at 12,000 *g* and at 4 °C for 10 min, the supernatants (crude nuclear extracts) were stored at -80 °C until further use [43,44]. Sirtuin-1 activity was determined in 50 micrograms of crude nuclear extracts from the study muscles in all the experimental groups. 

### 2.6. Statistical Analysis

All the statistical analyses were performed using STATA (software for Statistics and Data Science) software (StataCorp LLC, College Station, Texas, USA). The results are presented as mean values (standard deviation). Shapiro-Wilk test was used to test normality of the study variables. Results of the variables food intake and percentage of change of total body weight, limb strength, and muscle structure are represented in Table 1 and Table 2. The biological variables are represented in Figures. Two-way analysis of variance (ANOVA) was used to analyze the following effects: immobilization/recovery, treatment with curcumin, and interaction between these factors for all the study variables. Moreover, potential differences between two groups were analyzed using contrast of marginal linear predictions. Three levels of comparisons were established for all the study variables: 1) comparisons between recovery and immobilized mice, 2) comparisons between 7dI+Curcumin and 7dI, and 3) comparisons between the 7dR+curcumin mice and the 7dR rodents. *P* ≤ 0.05 was established as the level of significance.

## 3. Results

### 3.1. Physiological Characteristics of the Study Animals

*Recovery versus immobilization conditions*. Daily food intake did not significantly differ between immobilized and recovery animals (Table 1). Total body weight significantly improved (*p* = 0.000) in the recovery mice compared to unloading animals (Table 1). No significantly differences were seen in the weight of the gastrocnemius between recovery and unloading animals (Table 1). Limb strength gain significantly improved (*p* = 0.001) in the recovery animals compared to immobilized mice (Table 1). 

*Immobilization with curcumin versus immobilization*. No significant differences were seen in the variables: food intake, total body and gastrocnemius weight, or limb strength gain between unloading mice and those treated with curcumin (Table 1). 

*Recovery with curcumin versus recovery*. No significant differences were seen in the variables: food intake, total body and gastrocnemius weight, or limb strength gain between recovery mice and those treated with curcumin (Table 1). 

### 3.2. Structural Phenotypic Characteristics

*Recovery versus immobilization conditions*. No significant differences were observed in the proportions of either slow- or fast-twitch fiber types in the gastrocnemius among the study groups (Table 2 and Figure 2). Compared to non-treated immobilized mice, in the limb muscle of the recovery animals, CSA of type II fibers almost significantly increased (*p* = 0.081, Table 2 and Figure 2). CSA of slow-twitch and hybrid fibers did not significantly differ between these two groups of mice (Table 2 and Figure 2). The number of TUNEL-positive nuclei significantly decreased (*p* = 0.000) in the gastrocnemius of the reloading animals (Table 2 and Figure 3). 

*Immobilization with curcumin versus immobilization*. No significant differences were detected in the proportions or size of the slow- or fast-twitch fibers or hybrid fibers in the gastrocnemius between unloading and curcumin-treated unloading mice (Table 2 and Figure 2). Importantly, TUNEL-positive nuclei were significantly lower (*p* = 0.000) in the muscles of the unloading animals treated with curcumin compared to the non-treated mice (Table 2 and Figure 3). 

*Recovery with curcumin versus recovery*. No significant differences were detected in the proportions or size of the slow- or fast-twitch fibers or hybrid fibers in the gastrocnemius between recovery and curcumin-treated reloading mice (Table 2 and Figure 2). The size of the hybrid fibers was significantly greater (*p* = 0.030) in the gastrocnemius of the recovery mice treated with curcumin than in the non-treated recovery animals (Table 2 and Figure 2). The number of TUNEL-positive nuclei significantly decreased (*p* = 0.024) in the muscles of the recovery animals treated with curcumin (Table 2 and Figure 3). 

### 3.3. Sirtuin-1 Content of and Activity 

**Figure 4:**
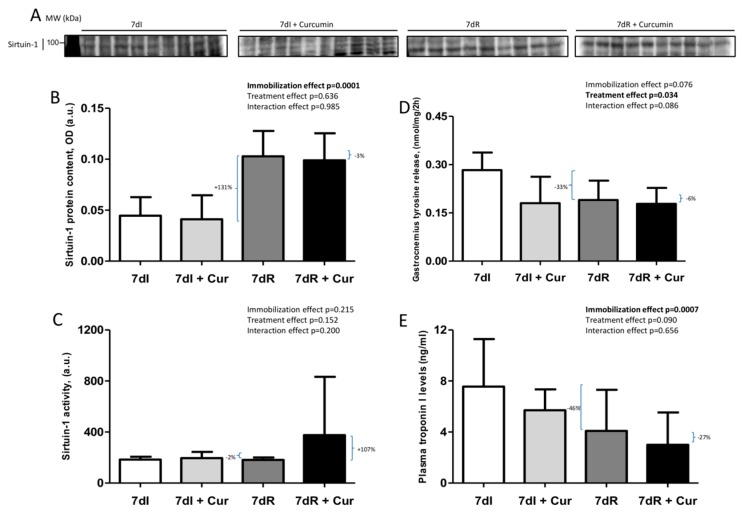
(**A**) Representative immunoblots of sirtuin-1 protein in the gastrocnemius muscle of all study groups of mice. Definition of abbreviations: MW, molecular weight; kDa, kilodalton; I, immobilization; R, recovery. (**B**) Mean values and standard deviation of sirtuin-1 protein content in the gastrocnemius muscle of the different study groups of mice, as measured by optical densities in arbitrary units (OD, a.u.). Definition of abbreviations: OD, optical densities; a.u., arbitrary units; I, immobilization; R, recovery; Cur, curcumin. The statistical analyses (two-way ANOVA test) of the effect of immobilization, treatment and interaction effects, are also indicated as actual P values for each variable. The percentage of change between: 1) 7dR and 7dI and 2) 7dR + Curcumin and 7dR are also indicated for each variable. (**C**) Mean values and standard deviation of sirtuin-1 activity levels in the gastrocnemius muscle of the different study groups of mice, as measured by fluorescence in arbitrary units (a.u.). Definition of abbreviations: a.u., arbitrary units; I, immobilization; R, recovery; Cur, curcumin. The statistical analyses (two-way ANOVA test) of the effect of immobilization, treatment and interaction effects, are also indicated as actual P values for each variable. The percentage of change between: 1) 7dR and 7dI and 2) 7dR + Curcumin and 7dR are also indicated for each variable. (**D**) Mean values and standard deviation of the variable tyrosine release (nmol/mg/2 h) of the gastrocnemius muscle of the different study groups of mice. Definition of abbreviations: nmol, nanomol; mg, milligram; h, hour; I, immobilization; R, recovery; Cur, curcumin. The statistical analyses (two-way ANOVA test) of the effect of immobilization, treatment and interaction effects, are also indicated as actual P values for each variable. The percentage of change between: 1) 7dR and 7dI and 2) 7dR + Curcumin and 7dR are also indicated for each variable. (**E**) Mean values and standard deviation of the variable plasma troponin-I (ng/ml) of the gastrocnemius muscle of the different study groups of mice. Definition of abbreviations: ng, nanogram; ml, milliliter; h, hour; I, immobilization; R, recovery; Cur, curcumin. The statistical analyses (two-way ANOVA test) of the effect of immobilization, treatment and interaction effects, are also indicated as actual P values for each variable. The percentage of change between: 1) 7dR and 7dI and 2) 7dR + Curcumin and 7dR are also indicated for each variable.

*Recovery versus immobilization conditions*. Compared to non-treated immobilized mice, in the muscles of the recovery animals, sirtuin-1 protein levels significantly improved (*p* = 0.001), but not those of sirtuin-1 activity (Figure 4A–C and Figure S3). 

*Immobilization with curcumin versus immobilization*. No significant differences were observed in either sirtuin-1 protein content or activity in the gastrocnemius between 7dI+Curcumin and 7dI mice (Figure 4A–C and Figure S3). 

*Recovery with curcumin versus recovery*. Compared to non-treated recovery animals, in gastrocnemius muscles of the treated mice, sirtuin-1 activity almost significantly increased (*p* = 0.116), while no significant differences were observed in sirtuin-1 content between the two groups (Figure 4A–C and Figure S3). 

### 3.4. Muscle Proteolysis

*Recovery versus immobilization conditions*. Proteolysis as measured by muscle tyrosine release (*p* = 0.009) and plasma troponin I levels (*p* = 0.006) were lower in the gastrocnemius of the recovery mice than the immobilized animals (Figure 4D,E). 

*Immobilization with curcumin versus immobilization*. Muscle proteolysis as measured by tyrosine release, but not troponin I was significantly reduced (*p* = 0.011) in the limb muscle of the immobilized mice treated with curcumin compared to the non-treated animals (Figure 4D,E).

*Recovery with curcumin versus recovery*. The markers tyrosine release or troponin I did not significantly differ between 7dR+Curcumin and 7dR animals (Figure 4D,E). 

### 3.5. Markers of Proteolysis

**Figure 5:**
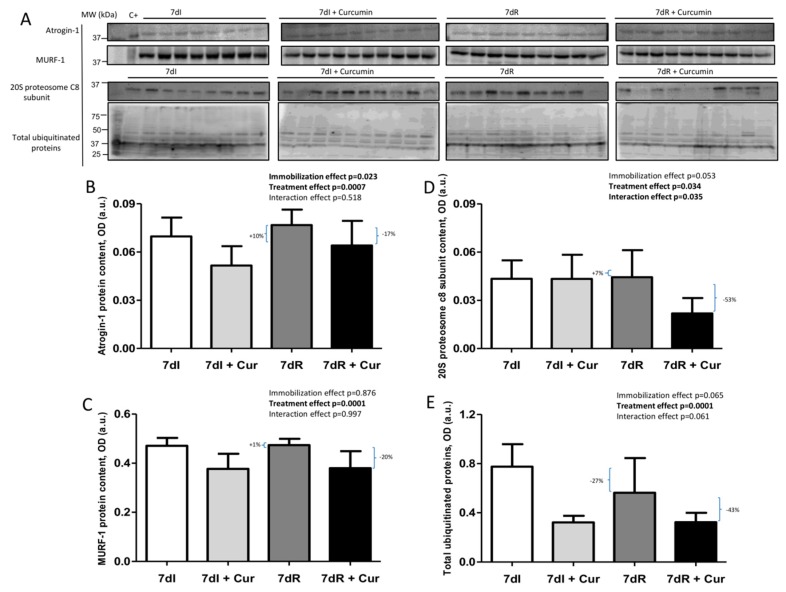
(**A**) Representative immunoblots of atrogin-1, MuRF-1, 20S proteasome alpha subunit C8 and total ubiquitinated proteins in the gastrocnemius muscle of all study groups of mice. Definition of abbreviations: MuRF-1, muscle RING-finger protein-1; C+, positive control; MW, molecular weight; kDa, kilodalton; I, immobilization; R, recovery. (**B**) Mean values and standard deviation of atrogin-1 protein content in the gastrocnemius muscle of the different study groups of mice, as measured by optical densities in arbitrary units (OD, a.u.). Definition of abbreviations: OD, optical densities; a.u., arbitrary units; I, immobilization; R, recovery; Cur, curcumin. The statistical analyses (two-way ANOVA test) of the effect of immobilization, treatment and interaction effects, are also indicated as actual P values for each variable. The percentage of change between: 1) 7dR and 7dI and 2) 7dR + Curcumin and 7dR are also indicated for each variable. (**C**) Mean values and standard deviation of MuRF-1 protein content in the gastrocnemius muscle of the different study groups of mice, as measured by optical densities in arbitrary units (OD, a.u.). Definition of abbreviations: MuRF-1, muscle ring finger protein 1; OD, optical densities; a.u., arbitrary units; I, immobilization; R, recovery; Cur, curcumin. The statistical analyses (two-way ANOVA test) of the effect of immobilization, treatment and interaction effects, are also indicated as actual P values for each variable. The percentage of change between: 1) 7dR and 7dI and 2) 7dR + Curcumin and 7dR are also indicated for each variable. (**D**) Mean values and standard deviation of 20S proteasome alpha subunit C8 protein content in the gastrocnemius muscle of the different study groups of mice, as measured by optical densities in arbitrary units (OD, a.u.). Definition of abbreviations: OD, optical densities; a.u., arbitrary units; I, immobilization; R, recovery; Cur, curcumin. The statistical analyses (two-way ANOVA test) of the effect of immobilization, treatment and interaction effects, are also indicated as actual P values for each variable. The percentage of change between: 1) 7dR and 7dI and 2) 7dR + Curcumin and 7dR are also indicated for each variable. (**E**) Mean values and standard deviation of total ubiquitinated proteins content in the gastrocnemius muscle of the different study groups of mice, as measured by optical densities in arbitrary units (OD, a.u.). Definition of abbreviations: OD, optical densities; a.u., arbitrary units; I, immobilization; R, recovery; Cur, curcumin. The statistical analyses (two-way ANOVA test) of the effect of immobilization, treatment and interaction effects, are also indicated as actual P values for each variable. The percentage of change between: 1) 7dR and 7dI and 2) 7dR + Curcumin and 7dR are also indicated for each variable.

*Recovery versus immobilization conditions*. Recovery elicited a significant decline (*p* = 0.032) in levels of muscle atrogin-1 along with an almost significant decrease (*p* = 0.053) in proteasome content and total protein ubiquitination (*p* = 0.005) but not MuRF-1 compared to immobilized animals (Figure 5A–E and Figures S4–S7). 

*Immobilization with curcumin versus immobilization*. Treatment with curcumin of the immobilized mice induced a significant decrease (*p* = 0.004) in muscle atrogin-1, MuRF-1 (*p* = 0.003), and total protein ubiquitination (*p* = 0.000), but not proteasome content compared to 7dI mice (Figure 5A–E and Figures S4–S7). 

*Recovery with curcumin versus recovery*. In gastrocnemius of 7dR+curcumin a significant decline (*p* = 0.033) in levels of atrogin-1, MuRF-1 (*p* = 0.004), proteasome content (*p* = 0.002), and total protein ubiquitination (*p* = 0.059) was detected compared to non-treated recovery mice (Figure 5A–E and Figures S4–S7). 

### 3.6. Muscle Specific Proteins and Markers of Protein Synthesis 

**Figure 6:**
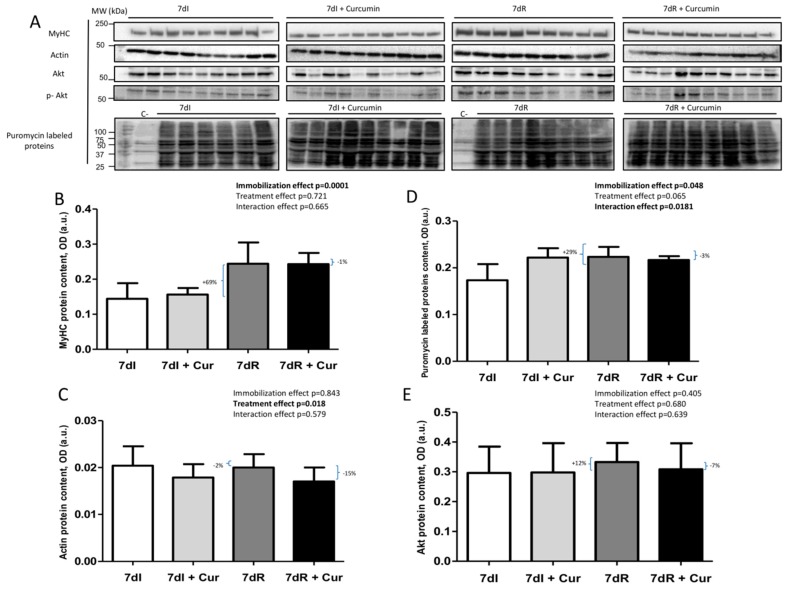

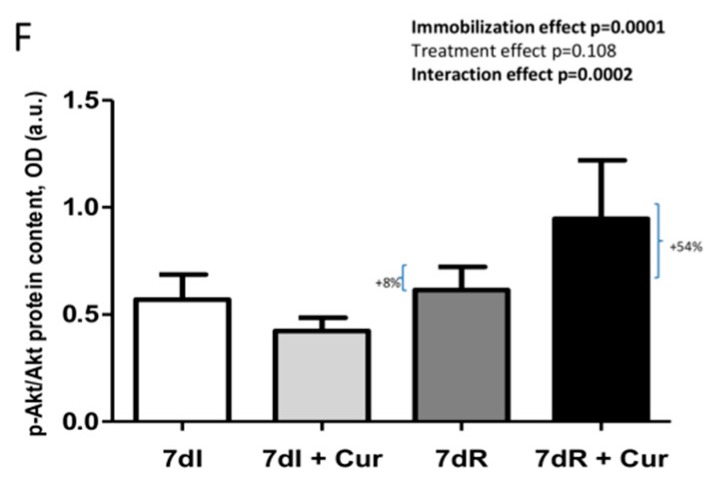
(**A**) Representative immunoblots of MyHC, actin, puromycin-labeled proteins, Akt and phosphorylated Akt proteins in the gastrocnemius muscle of all study groups of mice. Definition of abbreviations: MyHC, myosin heavy chain; Akt, Serine/Threonine Kinase 1, p-, phosphorylated; C-, negative control; MW, molecular weight; kDa, kilodalton; I, immobilization; R, recovery. (**B**) Mean values and standard deviation of MyHC protein content in the gastrocnemius muscle of the different study groups of mice, as measured by optical densities in arbitrary units (OD, a.u.). Definition of abbreviations: MyHC, myosin heavy chain; OD, optical densities; a.u., arbitrary units; I, immobilization; R, recovery; Cur, curcumin. The statistical analyses (two-way ANOVA test) of the effect of immobilization, treatment and interaction effects, are also indicated as actual P values for each variable. The percentage of change between: 1) 7dR and 7dI and 2) 7dR + Curcumin and 7dR are also indicated for each variable. (**C**) Mean values and standard deviation of actin protein content in the gastrocnemius muscle of the different study groups of mice, as measured by optical densities in arbitrary units (OD, a.u.). Definition of abbreviations: OD, optical densities; a.u., arbitrary units; I, immobilization; R, recovery; Cur, curcumin. The statistical analyses (two-way ANOVA test) of the effect of immobilization, treatment and interaction effects, are also indicated as actual P values for each variable. The percentage of change between: 1) 7dR and 7dI and 2) 7dR + Curcumin and 7dR are also indicated for each variable. (**D**) Mean values and standard deviation of the puromycin-labeled proteins levels in the gastrocnemius muscle of the different study groups of mice, as measured by optical densities in arbitrary units (OD, a.u.). Definition of abbreviations: OD, optical densities; a.u., arbitrary units; I, immobilization; R, recovery; Cur, curcumin. The statistical analyses (two-way ANOVA test) of the effect of immobilization, treatment and interaction effects, are also indicated as actual P values for each variable. The percentage of change between: 1) 7dR and 7dI and 2) 7dR + Curcumin and 7dR are also indicated for each variable. (**E**) Mean values and standard deviation of the Akt proteins levels in the gastrocnemius muscle of the different study groups of mice, as measured by optical densities in arbitrary units (OD, a.u.). Definition of abbreviations: Akt, Serine/Threonine Kinase 1; OD, optical densities; a.u., arbitrary units; I, immobilization; R, recovery; Cur, curcumin. The statistical analyses (two-way ANOVA test) of the effect of immobilization, treatment and interaction effects, are also indicated as actual P values for each variable. The percentage of change between: 1) 7dR and 7dI and 2) 7dR + Curcumin and 7dR are also indicated for each variable. (**F**) Mean values and standard deviation of phosphorylated Akt proteins levels in the gastrocnemius muscle of the different study groups of mice, as measured by optical densities in arbitrary units (OD, a.u.). Definition of abbreviations: p, phosphorylated; Akt, Serine/Threonine Kinase 1; OD, optical densities; a.u., arbitrary units; I, immobilization; R, recovery; Cur, curcumin. The statistical analyses (two-way ANOVA test) of the effect of immobilization, treatment and interaction effects, are also indicated as actual P values for each variable. The percentage of change between: 1) 7dR and 7dI and 2) 7dR + Curcumin and 7dR are also indicated for each variable.

*Recovery versus immobilization conditions*. In limb muscle of recovery mice, a significant rise in the variables MyHC (*p* = 0.001), puromycin-labeled proteins (*p* = 0.002), and phosphorylated Akt (*p* = 0.051) was detected compared to immobilized mice (Figure 6A–F and Figures S8–S12). Actin and total Akt content did not differ between these two groups of animals (Figure 6A–F and Figures S8–S12).

*Immobilization with curcumin versus immobilization*. Curcumin, but not MyHC, actin total Akt, or phosphorylated Akt, elicited a significant rise in levels of puromycin-labeled proteins (*p* = 0.005) in the limb muscle of immobilized animals compared to non-treated mice (Figure 6A–F and Figures S8–S12).

*Recovery with curcumin versus recovery*. Levels of phosphorylated Akt significant increase (*p* = 0.000) in the limb muscle of the recovery mice treated with curcumin compared to non-treated animals, while no significant differences were seen in the variables MyHC, actin, puromycin-labeled proteins, or total Akt levels (Figure 6A–F and Figures S8–S12).

### 3.7. Muscle Atrophy Signaling Markers

**Figure 7:**
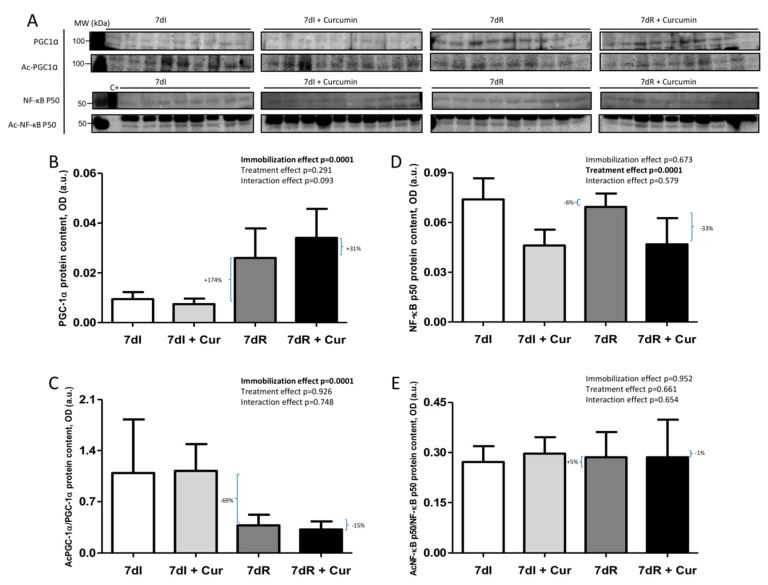
(**A**) Representative immunoblots of PGC-1α, acetylated PGC-1α, NF-κB p50 and acetylated NF-κB p50 proteins in the gastrocnemius muscle of all study groups of mice. Definition of abbreviations: PGC-1α, peroxisome proliferator-activated receptor gamma coactivator 1-alpha; NF-κB p50, nuclear factor kappa-light-chain-enhancer of activated B cells p50; ac-, acetylated; C+, positive control; MW, molecular weight; kDa, kilodalton; I, immobilization; R, recovery. (**B**) Mean values and standard deviation of PGC-1α protein content in the gastrocnemius muscle of the different study groups of mice, as measured by optical densities in arbitrary units (OD, a.u.). Definition of abbreviations: PGC-1α, peroxisome proliferator-activated receptor gamma coactivator 1-alpha; OD, optical densities; a.u., arbitrary units; I, immobilization; R, recovery; Cur, curcumin. The statistical analyses (two-way ANOVA test) of the effect of immobilization, treatment and interaction effects, are also indicated as actual P values for each variable. The percentage of change between: 1) 7dR and 7dI and 2) 7dR + Curcumin and 7dR are also indicated for each variable. (**C**) Mean values and standard deviation of acetylated PGC-1α protein content in the gastrocnemius muscle of the different study groups of mice, as measured by optical densities in arbitrary units (OD, a.u.). Definition of abbreviations: Ac, acetylated; PGC-1α, peroxisome proliferator-activated receptor gamma coactivator 1-alpha; OD, optical densities; a.u., arbitrary units; I, immobilization; R, recovery; Cur, curcumin. The statistical analyses (two-way ANOVA test) of the effect of immobilization, treatment and interaction effects, are also indicated as actual P values for each variable. The percentage of change between: 1) 7dR and 7dI and 2) 7dR + Curcumin and 7dR are also indicated for each variable. (**D**) Mean values and standard deviation of NF-κB p50 protein content in the gastrocnemius muscle of the different study groups of mice, as measured by optical densities in arbitrary units (OD, a.u.). Definition of abbreviations: NF-κB p50, nuclear factor kappa-light-chain-enhancer of activated B cells p50; OD, optical densities; a.u., arbitrary units; I, immobilization; R, recovery; Cur, curcumin. The statistical analyses (two-way ANOVA test) of the effect of immobilization, treatment and interaction effects, are also indicated as actual P values for each variable. The percentage of change between: 1) 7dR and 7dI and 2) 7dR + Curcumin and 7dR are also indicated for each variable. (E) Mean values and standard deviation of acetylated NF-κB p50 protein content in the gastrocnemius muscle of the different study groups of mice, as measured by optical densities in arbitrary units (OD, a.u.). Definition of abbreviations: Ac, acetylated; NF-κB p50, nuclear factor kappa-light-chain-enhancer of activated B cells p50; OD, optical densities; a.u., arbitrary units; I, immobilization; R, recovery; Cur, curcumin The statistical analyses (two-way ANOVA test) of the effect of immobilization, treatment and interaction effects, are also indicated as actual P values for each variable. The percentage of change between: 1) 7dR and 7dI and 2) 7dR + Curcumin and 7dR are also indicated for each variable.

**Figure 8:**
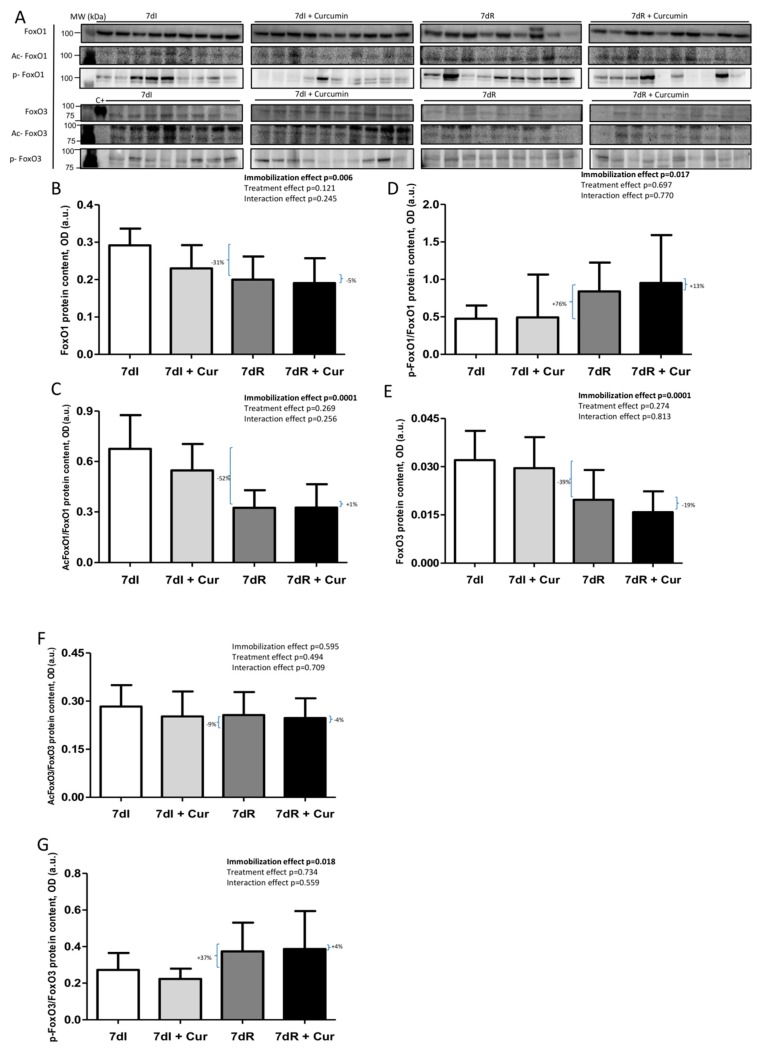
(**A**) Representative immunoblots of FoxO1, acetylated FoxO1, phosphorylated FoxO1, FoxO3, acetylated FoxO3 and phosphorylated FoxO3 proteins in the gastrocnemius muscle of all study groups of mice. Definition of abbreviations: FoxO1, transcription factor fork-head box O1; FoxO3, transcription factor fork-head box O3; ac-, acetylated; p-, phosphorylated; C+, positive control; MW, molecular weight; kDa, kilodalton; I, immobilization; R, recovery. (**B**) Mean values and standard deviation of FoxO1 protein content in the gastrocnemius muscle of the different study groups of mice, as measured by optical densities in arbitrary units (OD, a.u.). Definition of abbreviations: FoxO1, transcription factor fork-head box O1; OD, optical densities; a.u., arbitrary units; I, immobilization; R, recovery; Cur, curcumin. The statistical analyses (two-way ANOVA test) of the effect of immobilization, treatment and interaction effects, are also indicated as actual P values for each variable. The percentage of change between: 1) 7dR and 7dI and 2) 7dR + Curcumin and 7dR are also indicated for each variable. (**C**) Mean values and standard deviation of acetylated FoxO1 protein content in the gastrocnemius muscle of the different study groups of mice, as measured by optical densities in arbitrary units (OD, a.u.). Definition of abbreviations: Ac, acetylated; FoxO1, transcription factor fork-head box O1; OD, optical densities; a.u., arbitrary units; I, immobilization; R, recovery; Cur, curcumin. The statistical analyses (two-way ANOVA test) of the effect of immobilization, treatment and interaction effects, are also indicated as actual P values for each variable. The percentage of change between: 1) 7dR and 7dI and 2) 7dR + Curcumin and 7dR are also indicated for each variable. (**D**) Mean values and standard deviation of phosphorylated FoxO1 protein content in the gastrocnemius muscle of the different study groups of mice, as measured by optical densities in arbitrary units (OD, a.u.). Definition of abbreviations: p, phosphorylated; FoxO1, transcription factor fork-head box O1; OD, optical densities; a.u., arbitrary units; I, immobilization; R, recovery; Cur, curcumin. The statistical analyses (two-way ANOVA test) of the effect of immobilization, treatment and interaction effects, are also indicated as actual P values for each variable. The percentage of change between: 1) 7dR and 7dI and 2) 7dR + Curcumin and 7dR are also indicated for each variable. (**E**) Mean values and standard deviation of FoxO3 protein content in the gastrocnemius muscle of the different study groups of mice, as measured by optical densities in arbitrary units (OD, a.u.). Definition of abbreviations: FoxO3, transcription factor fork-head box O3; OD, optical densities; a.u., arbitrary units; I, immobilization; R, recovery; Cur, curcumin. The statistical analyses (two-way ANOVA test) of the effect of immobilization, treatment and interaction effects, are also indicated as actual P values for each variable. The percentage of change between: 1) 7dR and 7dI and 2) 7dR + Curcumin and 7dR are also indicated for each variable. (**F**) Mean values and standard deviation of acetylated FoxO3 protein content in the gastrocnemius muscle of the different study groups of mice, as measured by optical densities in arbitrary units (OD, a.u.). Definition of abbreviations: FoxO3, transcription factor fork-head box O3; OD, optical densities; a.u., arbitrary units; I, immobilization; R, recovery; Cur, curcumin. The statistical analyses (two-way ANOVA test) of the effect of immobilization, treatment and interaction effects, are also indicated as actual P values for each variable. The percentage of change between: 1) 7dR and 7dI and 2) 7dR + Curcumin and 7dR are also indicated for each variable. (**G**) Mean values and standard deviation of phosphorylated FoxO3 protein content in the gastrocnemius muscle of the different study groups of mice, as measured by optical densities in arbitrary units (OD, a.u.). Definition of abbreviations: p, phosphorylated; FoxO3, transcription factor fork-head box O3; OD, optical densities; a.u., arbitrary units; I, immobilization; R, recovery; Cur, curcumin. The statistical analyses (two-way ANOVA test) of the effect of immobilization, treatment and interaction effects, are also indicated as actual P values for each variable. The percentage of change between: 1) 7dR and 7dI and 2) 7dR + Curcumin and 7dR are also indicated for each variable.

*Recovery versus immobilization conditions*. Recovery induced in the limb muscle a significant rise (*p* = 0.024) in total PGC-1alpha levels along with a decline (*p* = 0.001) in the acetylation levels of this transcription factor, while no significant differences were seen in levels of NF-kB p50 subunit (Figure 7A–E and Figures S13–S16). Protein levels of FoxO1 and acetylated FoxO1 significantly declined (*p* = 0.052 and *p* = 0.002, respectively), while those of phosphorylated FoxO1 increased (*p* = 0.040) in the limb muscle of the recovery mice compared to immobilized animals (Figure 8A–D and Figures S17–S19). In the gastrocnemius of the recovery mice compared to immobilized animals, total FoxO3 levels decreased (*p* = 0.006), while those of phosphorylated increased (*p* = 0.020), and those of acetylated FoxO3 did not differ (Figure 8A,E–G, and Figures S20–S22). 

*Immobilization with curcumin versus immobilization*. Compared to non-treated 7dI mice, treatment with curcumin of the immobilized animals elicited a significant decrease (*p* = 0.002) in total NF-kB p50 subunit in the limb muscle, with no significant differences in the markers PGC-1alpha (total and acetylated) or acetylated NF-kB p50 (Figure 7A–E, and Figures S13–S16). Total levels of FoxO1 (*p* = 0.020) and those of acetylated FoxO1 (*p* = 0.102) but not those of phosphorylated FoxO1 were lower in the gastrocnemius of the immobilized mice treated with curcumin than in the non-treated animals (Figure 8A–D and Figures S17–S19). Muscle levels of total FoxO3, acetylated FoxO3 or those of phosphorylated FoxO3 did not differ between 7dI+Curcumin and 7dI animals (Figure 8A,E–G, and Figures S20–S22). 

*Recovery with curcumin versus recovery*. Compared to non-treated recovery mice, curcumin elicited an almost significant increase (*p* = 0.065) in total muscle PGC-1alpha levels of the recovery mice, while levels of NF-kB p50 subunit were significantly reduced (*p* = 0.003), and those of acetylated PGC-1alpha or acetylated NF-kB p50 did not differ between the two groups (Figure 7A–E, and Figures S13–S16). No significant differences were seen in muscle levels of total FoxO1, FoxO3, acetylated FoxO1, acetylated FoxO3, phosphorylated FoxO1, or phosphorylated FoxO3 between recovery animals treated with curcumin and those without the treatment (Figure 8A–G and Figures S17–S22). 

### 3.8. Histone Deacetylases in Muscles

**Figure 9:**
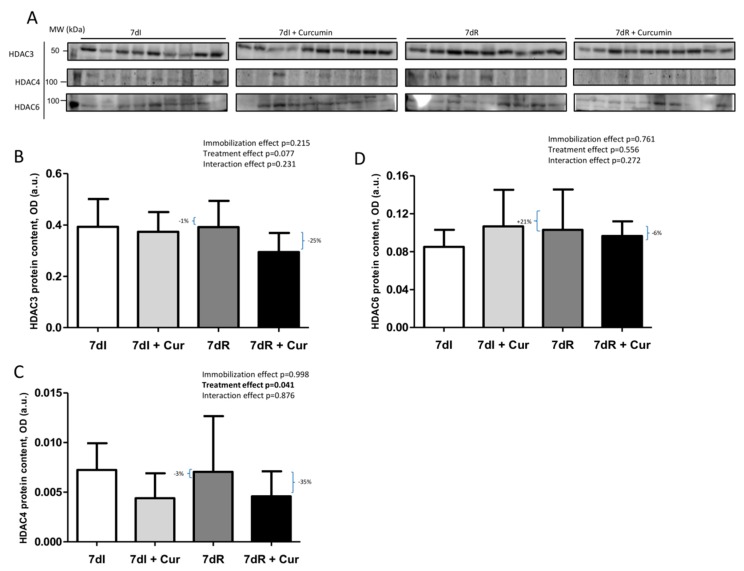
(**A**) Representative immunoblots of HDAC3, HDAC4 and HDAC6 proteins in the gastrocnemius muscle of all study groups of mice. Definition of abbreviations: HDAC3, histone deacetylase 3; HDAC4, histone deacetylase 3; HDAC6, histone deacetylase 6; MW, molecular weight; kDa, kilodalton; I, immobilization; R, recovery. (**B**) Mean values and standard deviation of HDAC3 protein content in the gastrocnemius muscle of the different study groups of mice, as measured by optical densities in arbitrary units (OD, a.u.). Definition of abbreviations: HDAC3, Histone deacetylase 3; OD, optical densities; a.u., arbitrary units; I, immobilization; R, recovery; Cur, curcumin. The statistical analyses (two-way ANOVA test) of the effect of immobilization, treatment and interaction effects, are also indicated as actual P values for each variable. The percentage of change between: 1) 7dR and 7dI and 2) 7dR + Curcumin and 7dR are also indicated for each variable. (**C**) Mean values and standard deviation of HDAC4 protein content in the gastrocnemius muscle of the different study groups of mice, as measured by optical densities in arbitrary units (OD, a.u.). Definition of abbreviations: HDAC4, Histone deacetylase 4; OD, optical densities; a.u., arbitrary units; I, immobilization; R, recovery; Cur, curcumin. The statistical analyses (two-way ANOVA test) of the effect of immobilization, treatment and interaction effects, are also indicated as actual P values for each variable. The percentage of change between: 1) 7dR and 7dI and 2) 7dR + Curcumin and 7dR are also indicated for each variable. (**D**) Mean values and standard deviation of HDAC6 protein content in the gastrocnemius muscle of the different study groups of mice, as measured by optical densities in arbitrary units (OD, a.u.). Definition of abbreviations: HDAC6, Histone deacetylase 6; OD, optical densities; a.u., arbitrary units; I, immobilization; R, recovery; Cur, curcumin. The statistical analyses (two-way ANOVA test) of the effect of immobilization, treatment and interaction effects, are also indicated as actual P values for each variable. The percentage of change between: 1) 7dR and 7dI and 2) 7dR + Curcumin and 7dR are also indicated for each variable.

*Recovery versus immobilization conditions*. No significant differences were seen in either HDAC3, HDAC4, or HDAC6 protein levels in the gastrocnemius between recovery and immobilized animals (Figure 9A–D and Figures S23–S25). 

*Immobilization with curcumin versus immobilization*. Protein levels of HDAC4, but not those of HDAC3 or HDAC6 significantly decreased (*p* = 0.038) in the limb muscle of the 7dI+Curcumin compared to 7dI mice (Figure 9A–D and Figures S23–S25). 

*Recovery with curcumin versus recovery*. Protein levels of HDAC3, but not those of HDAC4 or HDAC6 significantly declined (*p* = 0.030) in the limb muscle of the 7dR+Curcumin compared to 7dR mice (Figure 9A–D and Figures S23–S25). 

## 4. Discussion

Disuse muscle atrophy is relevant in patients with chronic conditions and during prolonged bed rest due to surgical interventions, critical illness, trauma, and acute exacerbations of chronic conditions. Moreover, those scenarios may coincide in the same patient. On the other hand, disuse muscle atrophy may also aggravate sarcopenia and/or cachexia of the patients, especially of those with systemic manifestations of their organ disease such as chronic heart and lung diseases. 

In the current investigation, in the gastrocnemius of recovery mice compared to immobilized animals, body weight, limb grip strength, CSA of fast-twitch fibers, sirtuin-1 and MyHC protein levels, puromycin-labeled proteins, phosphorylated Akt levels, PGG-1alpha, and phosphorylated FoxO1 and FoxO3 significantly increased, while numbers of TUNEL-positive nuclei, muscle proteolysis and specific markers of proteolysis, acetylated PGC-1alpha and FoxO1, and total FoxO1 and FoxO3 significantly decreased. 

In the limb muscle of immobilized animals treated with curcumin compared to non-treated immobilized mice, puromycin-labeled proteins significantly increased, whereas numbers of TUNEL-positive nuclei, tyrosine release, markers of proteolysis, NF-kB p50 subunit, total FoxO1, acetylated FoxO1, and HDAC4 protein levels were significantly reduced.

In the gastrocnemius muscle of recovery mice treated with curcumin compared to non-treated recovery animals, CSA of hybrid fibers, sirtuin-1 activity, phosphorylated Akt, and total PGC-1alpha significantly increased, while numbers of TUNEL-positive nuclei, markers of muscle proteolysis, NF-kB p50 subunit, and HDAC3 protein levels significantly declined. The most relevant findings encountered in the investigation are discussed below. 

A larger decline in TUNEL-positive nuclei was seen between the two immobilized groups of mice (25% reduction between 7dI+Curcumin and 7dI) than between the two recovery groups (8%). A significant decline in TUNEL-positive nuclei was also observed in the recovery mice compared to the immobilized animals (31%). These findings suggest that curcumin elicited a larger beneficial effect on the immobilized muscles than in the recovery ones probably as a result of the relatively low levels of TUNEL-positive nuclei already detected in the non-treated recovery. 

A significant increase in protein synthesis as measured by puromycin-labeled proteins was also observed in the limb muscles of the immobilized control mice treated with curcumin. These findings suggest that curcumin somehow contributes to preventing proteolysis, while favoring protein synthesis in the control muscles in the current experimental model. The main findings reported herein are further discussed below. 

As previously demonstrated [23,24,25], a seven-day period of reloading induced beneficial effects on skeletal muscles that had been exposed to unloading for another seven-day period. Reloading of the hindlimb muscles for seven days favored body weight gain and limb muscle strength in the mice through several key biological mechanisms and signaling pathways that are involved in muscle mass maintenance, especially muscle protein catabolism. In keeping with, markers of muscle proteolysis (atrogin-1, MuRF-1, proteasome content, and total protein ubiquitination) significantly decreased in the limb muscle of the mice treated with curcumin under both conditions (immobilization and recovery periods). These findings are consistent with the increase in the size of the hybrid fibers that was detected in the recovery mice treated with curcumin. Protein levels of HDAC4 and HDAC6 were not modified by curcumin in the muscles in any of the treated mice, whereas levels of the histone deacetylase sirtuin-1 were increased in the recovery muscles of mice that received concomitant treatment with curcumin. Indeed, a significant rise in sirtuin-1 activity was only seen in the reloaded animals treated with curcumin but not in those that did not receive this treatment. These findings imply that the beneficial effects observed in the limb muscle are associated to a large extent with surtuin-1 activity. 

Sirtuin-1 is a nicotinamide adenine dinucleotide (NAD)+ dependent histone deacetylase involved in several important biological processes such as DNA repair, cell survival, aging [45], and muscle proteolysis [24,46]. In previous investigations from our group, sirtuin-1 protein levels were shown to be reduced in muscles and myotubes of patients with COPD and severe muscle wasting [46,47] and in mice exposed to hindlimb unloading for several periods [24,25]. Furthermore, treatment of the cachectic myotubes with the phosphodiesterase-4 inhibitor roflumilast elicited a significant increase in sirtuin-1 expression levels that were also associated with an attenuation of muscle proteolysis (tyrosine release assay) and proteolytic markers [46]. In the current study, as previously shown [23,24,25] reloading following a seven-day period of unloading induced a significant increase in sirtuin-1 protein levels in the gastrocnemius of the study mice. Importantly, concomitant treatment of the rodents with curcumin induced a rise in sirtuin-1 content in the limb muscle. The histone deacetylase sirtuin-1 is a key enzyme in cellular processes such as senescence of tissues, cell survival, antioxidative properties, cardiovascular disease and muscle wasting conditions [27]. In the present investigation, it is possible to conclude that attenuation of proteolysis and the recovery of muscle structure and function seen in the gastrocnemius of reloaded mice treated with curcumin was mediated by sirtuin-1 activity to a great extent. 

Curcumin, which is the active component of *Curcuma longa*, exerts beneficial effects in cells through attenuation of key processes such as inflammation, apoptosis, and oxidative stress among others [48]. Curcumin treatment was also shown to protect against heart fibrosis following myocardial infarction via sirtuin-1 activation [27], senescence of vessels [28], oxidative stress and force recovery in muscles of aged rats [49], oxidative damage of mitochondria in ischemia-reperfusion models [48], and autophagy and apoptosis in myocytes exposed to hypoxia/reoxygenation conditions [50]. In all those models, a rise in sirtuin-1 levels was observed, thus leading to the conclusion that the histone deacetylase played a major role in the protection exerted by curcumin treatment in the different animal and cell models [27,28,45,48,49,50]. 

In the present investigation, expression levels of acetylated transcription factors were also analyzed in the limb muscle of reloaded mice with and without treatment with curcumin. As previously demonstrated [24,25], recovery of the limb muscles also induced a significant decline in acetylation levels of PGC-1alpha and FoxO1, and in total FoxO3 in the gastrocnemius of the mice in the current study. Importantly, a significant decline in the active forms of FoxO1 and FoxO3 was also observed in the limb muscles of the recovery animals. On the other hand, a significant rise in the active form of Akt and puromycin-labeled proteins (protein synthesis) was also seen in the recovery muscles in response to curcumin along with an increase in the expression of PGC-1alpha protein levels. Taken together, these findings suggest that curcumin’s beneficial effects on muscles are two-fold: 1) it promoted protein synthesis and 2) it induced a decline in muscle protein degradation via ubiquitin-proteasome pathway as also suggested previously [51,52] 

Additionally, treatment of the animals with curcumin elicited a significant reduction in levels of NF-kB p50 subunit. These findings imply that part of the beneficial effects seen in the muscles of the reloaded mice treated with the phenolic compound may have been mediated via a reduction in NF-kB activity. Interestingly, curcumin treatment also elicited a significant decline in NF-kB p50 subunit in the immobilized muscles of mice. Similar findings were reported in aged muscles, in which oxidative stress and redox-sensitive signaling pathways decreased in response to curcumin therapy [48,49,50].

### Study Limitations

Differences in experimental models, mouse strains, duration of the protocols (acute versus chronic), and type of study muscles may account for differences in the levels of certain markers analyzed in the investigation in contrast with previous reports [53]. Moreover, differences in the methodological procedures to preserve the muscle samples may account for certain difficulties to identify all the MyHC isoforms as compared to other studies [54].

## 5. Conclusions

Treatment with the phenolic compound curcumin elicited a rise in sirtuin-1 activity, while attenuating proteolysis in the gastrocnemius muscle of mice during reloading following a period of unloading. Curcumin attenuated muscle proteolysis probably via the activation of the histone deacetylase sirtuin-1, which also led a to a decrease in levels of atrophy signaling pathways. These findings offer an avenue of research in the design of therapeutic strategies in clinical settings of patients exposed to periods of disuse muscle atrophy. 

## Figures and Tables

**Table 1:** Physiological parameters in all experimental groups of mice.

Physiological Parameters	7dI (N = 10)	7dI + Curcumin (N = 10)	7dR (N = 10)	7dR + Curcumin (N = 10)	Immobilization Effect *p*-value	Treatment Effect *p*-value	Interaction Effect *p*-value
Food intake (g/24 h)	3.18 (0.24)	3.37 (0.09)	3.32 (0.23), +5%	3.34 (0.12), 1%	0.199	0.09	0.08
Total body weight gain (%)	−5.94 (2.92)	−5.00 (3.24)	+0.99 (2.86), +119%	+3.37 (4.16), +223%	**0.0001**	0.135	0.471
Gastrocnemius weight (g)	0.092 (0.010)	0.095 (0.014)	0.098 (0.010), +7%	0.100 (0.009), +2%	0.195	0.847	0.250
Limb strength gain (%)	−10.85 (12.57)	−2.04 (16.26)	+10.92 (11.47), +200%	+14.10 (10.28), +29%	**0.001**	0.253	0.588

Variables are presented as mean (standard deviation). Definition of abbreviations: I, immobilization; R, recovery; the statistical analyses (two-way ANOVA test) of the effect of immobilization, treatment and interaction effects, are also indicated as actual P values for each variable; the percentage of change between: 1) 7dR and 7dI and 2) 7dR + Curcumin and 7dR are also indicated for each variable.

**Table 2:** Structural characteristics of the gastrocnemius muscle in the study groups.

Structural Characteristics	7dI (N = 10)	7dI + Curcumin (N = 10)	7dR (N = 10)	7dR + Curcumin (N = 10)	Immobilization Effect (*p*-value)	Treatment Effect (*p*-value)	Interaction Effect (*p*-value)
**Muscle fiber type, %**							
Type I fibers	16.88 (2.70)	18.45 (5.50)	16.38 (6.78), **−3%**	15.12 (4.36), **−8%**	0.190	0.722	0.904
Type II fibers	83.12 (2.70)	81.55 (5.50)	83.62 (6.78), **+1%**	84.88 (4.36), **+2%**	0.310	0.963	0.455
**Muscle fiber size (CSA)**							
Cross-sectional area, type I fibers	872.28 (241.46)	843.59 (133.10)	911.63 (187.08), **+5%**	996.27 (274.75), **+9%**	0.223	0.717	0.467
Cross-sectional area, type II fibers	922.02 (212.89)	948.48 (115.00)	1079.92 (119.03), **+17%**	1120.61 (146.98), **+4%**	**0.0134**	0.591	0.908
Muscle hybrid fiber, %	3.55 (1.61)	2.60 (0.67)	3.23 (3.22), **−9%**	1.82 (1.27), **−43%**	0.461	0.589	0.683
Cross-sectional area, hybrid fibers	881.97 (270.88)	543.23 (180.00)	715.92 (187.11), **−19%**	948.60 (328.35), **+33%**	0.292	0.635	**0.0191**
Number of apoptotic nuclei (TUNEL) %	68.90 (5.58)	51.69 (5.34)	47.42 (3.36), **−31%**	43.68 (3.09), **−9%**	**0.0001**	**0.0001**	**0.0004**

Variables are presented as mean (standard deviation). Definition of abbreviations: I, immobilization; R, recovery. The statistical analyses (two-way ANOVA test) of the effect of immobilization, treatment and interaction effects, are also indicated as actual P values for each variable. The percentage of change between: 1) 7dR and 7dI and 2) 7dR + Curcumin and 7dR are also indicated for each variable.

## Supplementary Materials

The following are available online at https://www.mdpi.com/2072-6643/12/2/388/s1, **Figure S1:** Representative examples of the gastrocnemius muscle in animals. Myofibers were stained in green, type I in the left image, type II in the middle image and negative control (omission of the primary antibody) in the right image. Hybrid fibers (black asterisk in left image and white asterisk in the middle image) are seen in both stained images. Definition of abbreviations: MyHC myosin heavy chain; I, immobilization; R, recovery; **Figure S2:** Representative examples of dystrophin-stained fibers (left panel) and of TUNEL assay (right panel) in the gastrocnemius muscles; **Figure S3:** Representative immunoblots of Sirtuin-1 protein in the gastrocnemius muscle of all study groups of mice. Definition of abbreviations: MW, molecular weight; kDa, kilodalton; I, immobilization; R, recovery; **Figure S4:** Representative immunoblots of Atrogin-1 protein in the gastrocnemius muscle of all study groups of mice. Definition of abbreviations: C+, positive control; MW, molecular weight; kDa, kilodalton; I, immobilization; R, recovery; **Figure S5:** Representative immunoblots of MuRF-1 protein in the gastrocnemius muscle of all study groups of mice. Definition of abbreviations: C+, positive control; MuRF-1, muscle RING-finger protein-1; MW, molecular weight; kDa, kilodalton; I, immobilization; R, recovery; **Figure S6:** Representative immunoblots of 20S proteasome alpha subunit C8 protein in the gastrocnemius muscle of all study groups of mice. Definition of abbreviations: MW, molecular weight; kDa, kilodalton; I, immobilization; R, recovery; **Figure S7:** Representative immunoblots of total ubiquitinated proteins in the gastrocnemius muscle of all study groups of mice. Definition of abbreviations: MW, molecular weight; kDa, kilodalton; I, immobilization; R, recovery; **Figure S8:** Representative immunoblots of MyHC protein in the gastrocnemius muscle of all study groups of mice. Definition of abbreviations: MyHC, myosin heavy chain; MW, molecular weight; kDa, kilodalton; I, immobilization; R, recovery; **Figure S9:** Representative immunoblots of actin protein in the gastrocnemius muscle of all study groups of mice. Definition of abbreviations: MW, molecular weight; kDa, kilodalton; I, immobilization; R, recovery; **Figure S10:** Representative immunoblots of puromycin labeled proteins in the gastrocnemius muscle of all study groups of mice. Definition of abbreviations: C-, negative control; MW, molecular weight; kDa, kilodalton; I, immobilization; R, recovery; **Figure S11:** Representative immunoblots of Akt protein in the gastrocnemius muscle of all study groups of mice. Definition of abbreviations: Akt, Serine/Threonine Kinase 1; MW, molecular weight; kDa, kilodalton; I, immobilization; R, recovery; **Figure S12:** Representative immunoblots of phosphorylated Akt protein in the gastrocnemius muscle of all study groups of mice. Definition of abbreviations: phosphor, phosphorylated; Akt, Serine/Threonine Kinase 1; MW, molecular weight; kDa, kilodalton; I, immobilization; R, recovery; **Figure S13:** Representative immunoblots of PGC-1α protein in the gastrocnemius muscle of all study groups of mice. Definition of abbreviations: PGC-1α, peroxisome proliferator-activated receptor gamma coactivator 1-alpha; MW, molecular weight; kDa, kilodalton; I, immobilization; R, recovery; **Figure S14:** Representative immunoblots of acetylated PGC-1α protein in the gastrocnemius muscle of all study groups of mice. Definition of abbreviations: PGC-1α, peroxisome proliferator-activated receptor gamma coactivator 1-alpha; MW, molecular weight; kDa, kilodalton; I, immobilization; R, recovery; **Figure S15:** Representative immunoblots of NF-κB p50 protein in the gastrocnemius muscle of all study groups of mice. *Definition of abbreviation:* NF-κB p50, nuclear factor kappa-light-chain-enhancer of activated B cells p50; MW, molecular weight; kDa, kilodalton; I, immobilization; R, recovery; **Figure S16:** Representative immunoblots of acetylated NF-κB p50 protein in the gastrocnemius muscle of all study groups of mice. Definition of abbreviations: NF-κB p50, nuclear factor kappa-light-chain-enhancer of activated B cells p50; MW, molecular weight; kDa, kilodalton; I, immobilization; R, recovery; **Figure S17:** Representative immunoblots of FoxO1 protein in the gastrocnemius muscle of all study groups of mice. Definition of abbreviations: FoxO1, transcription factor fork-head box O1; MW, molecular weight; kDa, kilodalton; I, immobilization; R, recovery; **Figure S18:** Representative immunoblots of acetylated FoxO1 protein in the gastrocnemius muscle of all study groups of mice. Definition of abbreviations: FoxO1, transcription factor fork-head box O1; MW, molecular weight; kDa, kilodalton; I, immobilization; R, recovery; **Figure S19:** Representative immunoblots of phosphorylated FoxO1 protein in the gastrocnemius muscle of all study groups of mice. Definition of abbreviations: phospho, phosphorylated; FoxO1, transcription factor fork-head box O1; MW, molecular weight; kDa, kilodalton; I, immobilization; R, recovery; **Figure S20:** Representative immunoblots of FoxO3 protein in the gastrocnemius muscle of all study groups of mice. Definition of abbreviations: FoxO3, transcription factor fork-head box O3; MW, molecular weight; kDa, kilodalton; I, immobilization; R, recovery; **Figure S21:** Representative immunoblots of acetylated FoxO3 protein in the gastrocnemius muscle of all study groups of mice. Definition of abbreviations: FoxO3, transcription factor fork-head box O3; MW, molecular weight; kDa, kilodalton; I, immobilization; R, recovery; **Figure S22:** Representative immunoblots of phosphorylated FoxO3 protein in the gastrocnemius muscle of all study groups of mice. Definition of abbreviations: phospho, phosphorylated; FoxO3, transcription factor fork-head box O3; MW, molecular weight; kDa, kilodalton; I, immobilization; R, recovery; **Figure S23:** Representative immunoblots of HDAC3 protein in the gastrocnemius muscle of all study groups of mice. Definition of abbreviations: HDAC3, histone deacetylase 3; MW, molecular weight; kDa, kilodalton; I, immobilization; R, recovery; **Figure S24:** Representative immunoblots of HDAC4 protein in the gastrocnemius muscle of all study groups of mice. Definition of abbreviations: HDAC4, histone deacetylase 4; MW, molecular weight; kDa, kilodalton; I, immobilization; R, recovery; **Figure S25:** Representative immunoblots of HDAC6 protein in the gastrocnemius muscle of all study groups of mice. Definition of abbreviations: HDAC6, histone deacetylase 6; MW, molecular weight; kDa, kilodalton; I, immobilization; R, recovery; **Figure S26:** Representative immunoblots of GAPDH protein in the gastrocnemius muscle of all study groups of mice. Definition of abbreviations: GAPDH, glyceraldehyde 3-phosphate dehydrogenase; MW, molecular weight; kDa, kilodalton; I, immobilization; R, recovery.
