# Supplementary material for: Muscle Phenotype, Proteolysis, and Atrophy Signaling During Reloading in Mice: Effects of Curcumin on the Gastrocnemius
Source: Nutrients. 2020 Jan 31;12(2):388. doi: 10.3390/nu12020388 (PMC7071295; doi:10.3390/nu12020388)

ONLINE SUPLEMENTAL DATA

MUSCLE PHENOTYPE, PROTEOLYSIS, AND ATROPHY SIGNALING DURING RELOADING IN MICE: EFFECTS OF CURCUMIN ON THE GASTROCNEMIUS

Laura Mañas-García, Nuria Bargalló, Joaquim Gea, and Esther Barreiro

FIGURE LEGENDS

Figure S1: Representative examples of the gastrocnemius muscle in animals. Myofibers were stained in green, type I in the left image, type II in the middle image and negative control (omission of the primary antibody) in the right image. Hybrid fibers (black asterisk in left image and white asterisk in the middle image) are seen in both stained images. *Definition of abbreviations:* MyHC myosin heavy chain; I, immobilization; R, recovery.

Figure S2: Representative examples of dystrophin-stained fibers (left panel) and of TUNEL assay (right panel) in the gastrocnemius muscles.

Figure S3: Representative immunoblots of Sirtuin-1 protein in the gastrocnemius muscle of all study groups of mice. *Definition of abbreviations:* MW, molecular weight; kDa, kilodalton; I, immobilization; R, recovery.

Figure S4: Representative immunoblots of Atrogin-1 protein in the gastrocnemius muscle of all study groups of mice. *Definition of abbreviations:* C+, positive control; MW, molecular weight; kDa, kilodalton; I, immobilization; R, recovery.

Figure S5: Representative immunoblots of MuRF-1 protein in the gastrocnemius muscle of all study groups of mice. *Definition of abbreviations:* C+, positive control; MuRF-1, muscle RING-finger protein-1; MW, molecular weight; kDa, kilodalton; I, immobilization; R, recovery.

Figure S6: Representative immunoblots of 20S proteasome alpha subunit C8 protein in the gastrocnemius muscle of all study groups of mice. *Definition of abbreviations:* MW, molecular weight; kDa, kilodalton; I, immobilization; R, recovery.

Figure S7: Representative immunoblots of total ubiquitinated proteins in the gastrocnemius muscle of all study groups of mice. *Definition of abbreviations:* MW, molecular weight; kDa, kilodalton; I, immobilization; R, recovery.

Figure S8: Representative immunoblots of MyHC protein in the gastrocnemius muscle of all study groups of mice. *Definition of abbreviations:* MyHC, myosin heavy chain; MW, molecular weight; kDa, kilodalton; I, immobilization; R, recovery.

Figure S9: Representative immunoblots of actin protein in the gastrocnemius muscle of all study groups of mice. *Definition of abbreviations:* MW, molecular weight; kDa, kilodalton; I, immobilization; R, recovery.

Figure S10: Representative immunoblots of puromycin labeled proteins in the gastrocnemius muscle of all study groups of mice. *Definition of abbreviations:* C-, negative control; MW, molecular weight; kDa, kilodalton; I, immobilization; R, recovery.

Figure S11: Representative immunoblots of Akt protein in the gastrocnemius muscle of all study groups of mice. *Definition of abbreviations:* Akt, Serine/Threonine Kinase 1; MW, molecular weight; kDa, kilodalton; I, immobilization; R, recovery.

Figure S12: Representative immunoblots of phosphorylated Akt protein in the gastrocnemius muscle of all study groups of mice. *Definition of abbreviations:* phosphor, phosphorylated; Akt, Serine/Threonine Kinase 1; MW, molecular weight; kDa, kilodalton; I, immobilization; R, recovery.

Figure S13: Representative immunoblots of PGC-1α protein in the gastrocnemius muscle of all study groups of mice. *Definition of abbreviations:* PGC-1α, peroxisome proliferator-activated receptor gamma coactivator 1-alpha; MW, molecular weight; kDa, kilodalton; I, immobilization; R, recovery.

Figure S14: Representative immunoblots of acetylated PGC-1α protein in the gastrocnemius muscle of all study groups of mice. *Definition of abbreviations:* PGC-1α, peroxisome proliferator-activated receptor gamma coactivator 1-alpha; MW, molecular weight; kDa, kilodalton; I, immobilization; R, recovery.

Figure S15: Representative immunoblots of NF-κB p50 protein in the gastrocnemius muscle of all study groups of mice. *Definition of abbreviation:* NF-κB p50, nuclear factor kappa-light-chain-enhancer of activated B cells p50; MW, molecular weight; kDa, kilodalton; I, immobilization; R, recovery.

Figure S16: Representative immunoblots of acetylated NF-κB p50 protein in the gastrocnemius muscle of all study groups of mice. *Definition of abbreviations:* NF-κB p50, nuclear factor kappa-light-chain-enhancer of activated B cells p50; MW, molecular weight; kDa, kilodalton; I, immobilization; R, recovery.

Figure S17: Representative immunoblots of FoxO1 protein in the gastrocnemius muscle of all study groups of mice. *Definition of abbreviations:* FoxO1, transcription factor fork-head box O1; MW, molecular weight; kDa, kilodalton; I, immobilization; R, recovery.

Figure S18: Representative immunoblots of acetylated FoxO1 protein in the gastrocnemius muscle of all study groups of mice. *Definition of abbreviations:* FoxO1, transcription factor fork-head box O1; MW, molecular weight; kDa, kilodalton; I, immobilization; R, recovery.

Figure S19: Representative immunoblots of phosphorylated FoxO1 protein in the gastrocnemius muscle of all study groups of mice. *Definition of abbreviations:* phospho, phosphorylated; FoxO1, transcription factor fork-head box O1; MW, molecular weight; kDa, kilodalton; I, immobilization; R, recovery.

Figure S20: Representative immunoblots of FoxO3 protein in the gastrocnemius muscle of all study groups of mice. *Definition of abbreviations:* FoxO3, transcription factor fork-head box O3; MW, molecular weight; kDa, kilodalton; I, immobilization; R, recovery.

Figure S21: Representative immunoblots of acetylated FoxO3 protein in the

gastrocnemius muscle of all study groups of mice. *Definition of abbreviations:* FoxO3, transcription factor fork-head box O3; MW, molecular weight; kDa, kilodalton; I, immobilization; R, recovery.

Figure S22: Representative immunoblots of phosphorylated FoxO3 protein in the gastrocnemius muscle of all study groups of mice. *Definition of abbreviations:* phospho, phosphorylated; FoxO3, transcription factor fork-head box O3; MW, molecular weight; kDa, kilodalton; I, immobilization; R, recovery

Figure S23: Representative immunoblots of HDAC3 protein in the gastrocnemius muscle of all study groups of mice. *Definition of abbreviations:* HDAC3, histone deacetylase 3; MW, molecular weight; kDa, kilodalton; I, immobilization; R, recovery.

Figure S24: Representative immunoblots of HDAC4 protein in the gastrocnemius muscle of all study groups of mice. *Definition of abbreviations:* HDAC4, histone deacetylase 4; MW, molecular weight; kDa, kilodalton; I, immobilization; R, recovery.

Figure S25: Representative immunoblots of HDAC6 protein in the gastrocnemius muscle of all study groups of mice. *Definition of abbreviations:* HDAC6, histone deacetylase 6; MW, molecular weight; kDa, kilodalton; I, immobilization; R, recovery.

Figure S26: Representative immunoblots of GAPDH protein in the gastrocnemius muscle of all study groups of mice. *Definition of abbreviations:* GAPDH, glyceraldehyde 3-phosphate dehydrogenase; MW, molecular weight; kDa, kilodalton; I, immobilization; R, recovery.

Mañas-García L. et al. Figure S1

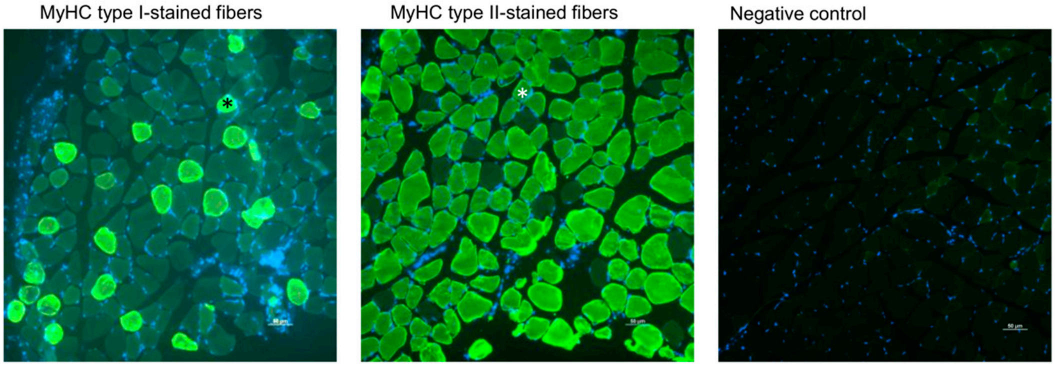

Mañas-García L. et al. Figure S2

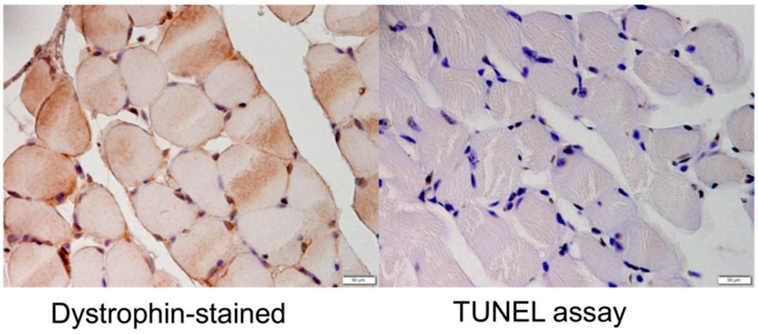

Mañas-García L. et al. Figure S3

Sirtuin-1 (100 kDa)

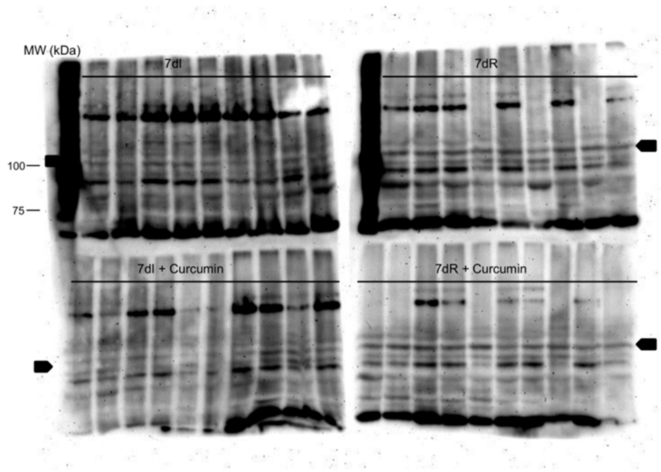

Mañas-García L. et al. Figure S4

Atrogin-1 (41 kDa)

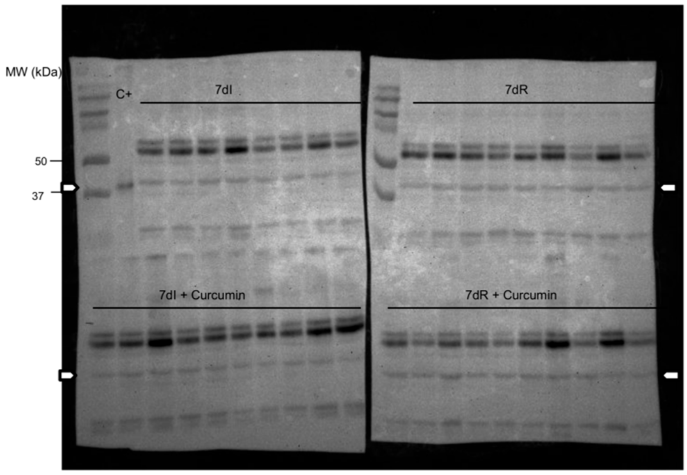

Mañas-García L. et al. Figure S5

MURF-1 (42 kDa)

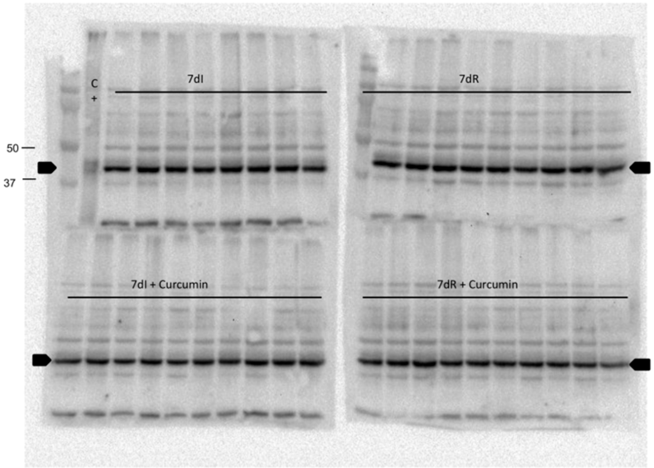

Mañas-García L. et al. Figure S6

20s Proteosome c8 subunit (30 kDa)

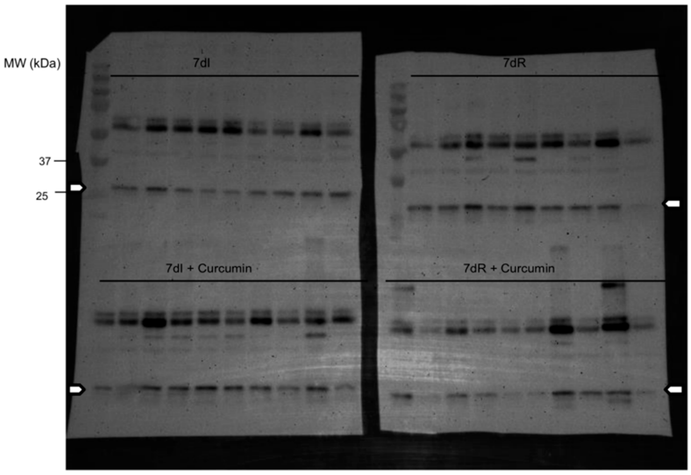

Mañas-García L. et al. Figure S7

Total ubiquitinated proteins

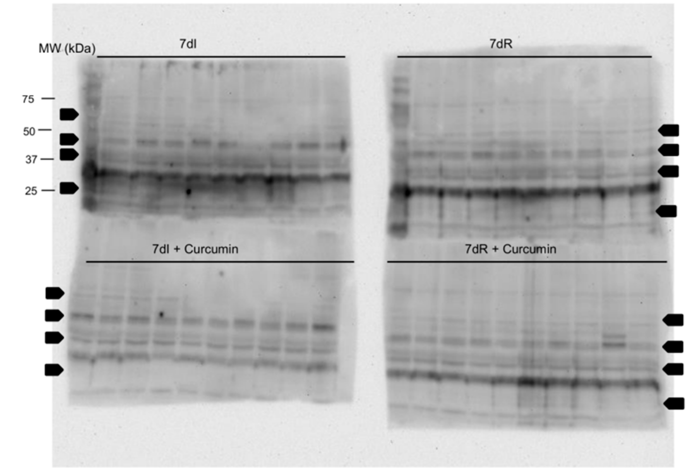

Mañas-García L. et al. Figure S8

MyHC (220 kDa)

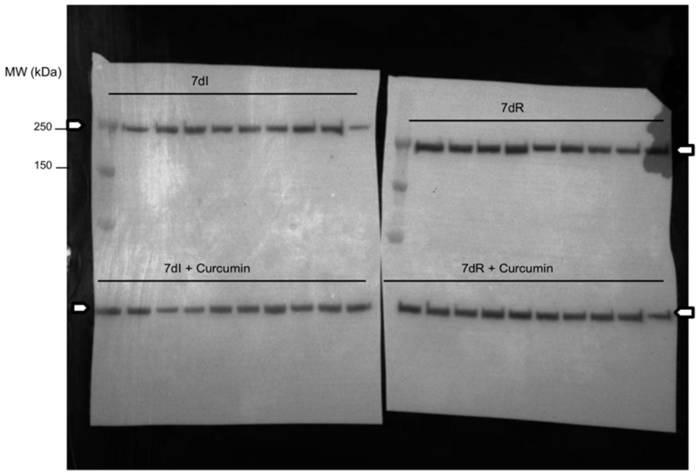

Mañas-García L. et al. Figure S19

Actin (43 kDa)

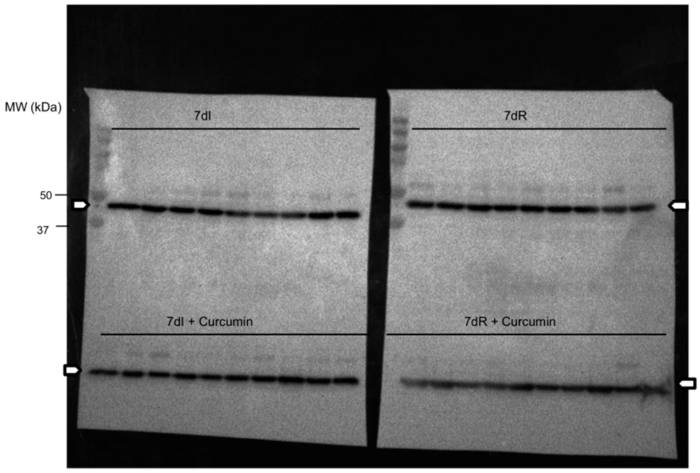

Mañas-García L. et al. Figure S10

Puromycin labeled proteins

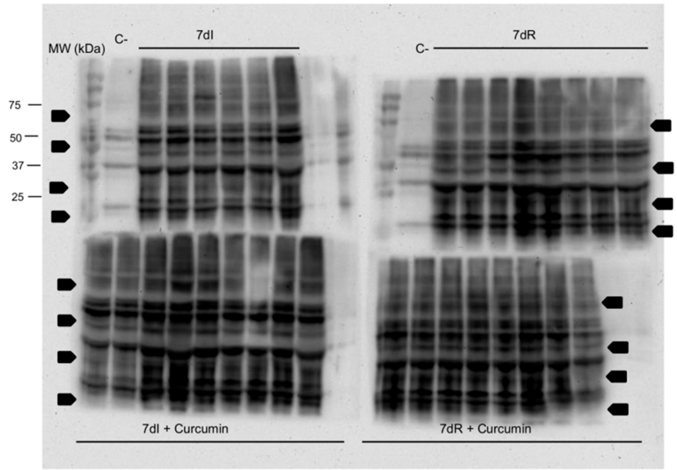

Mañas-García L. et al. Figure S12

Phospho-Akt (60 kDa)

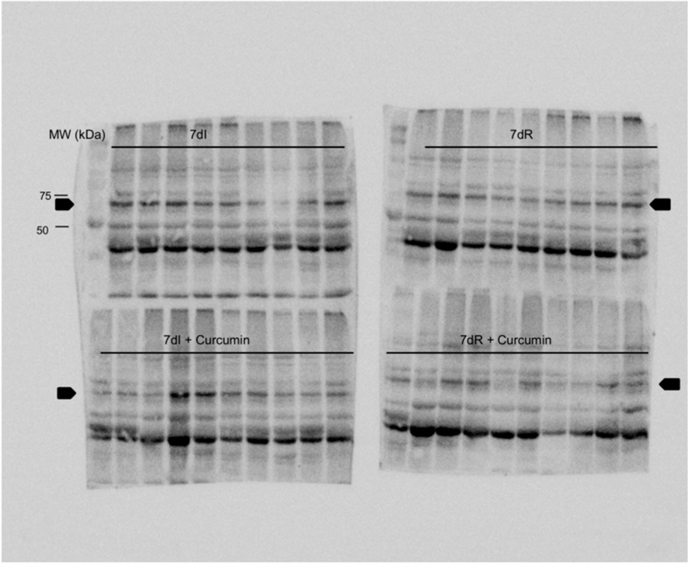

Mañas-García L. et al. Figure S13

PGC-1α (90 kDa)

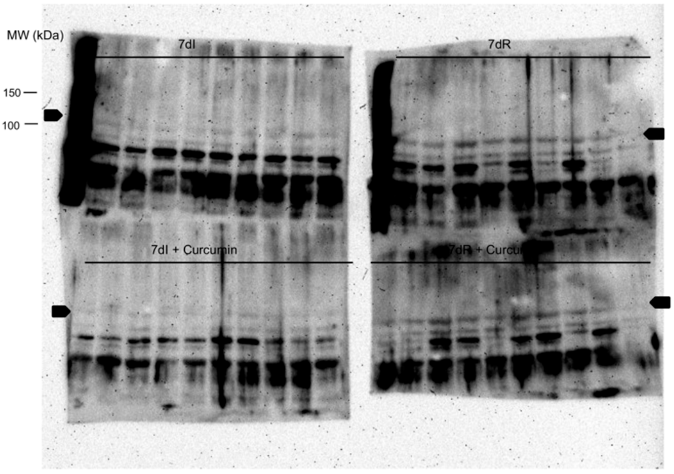

Mañas-García L. et al. Figure S11

Akt (60 kDa)

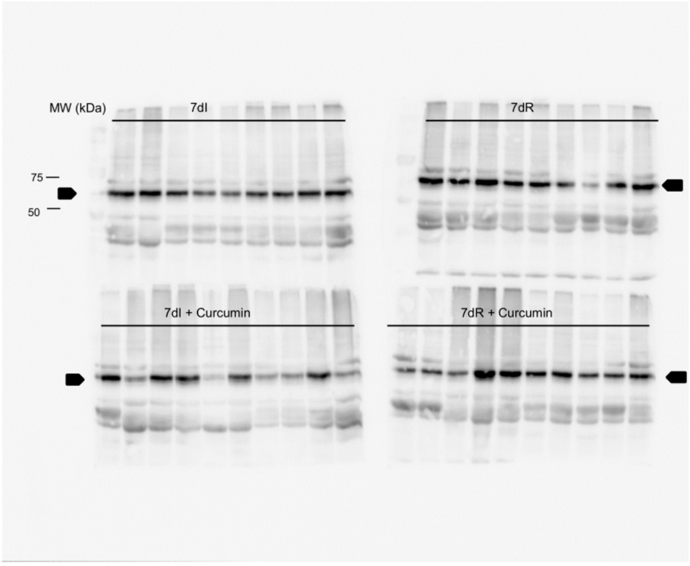

Mañas-García L. et al. Figure S14

Acetylated-PGC-1α (90 kDa)

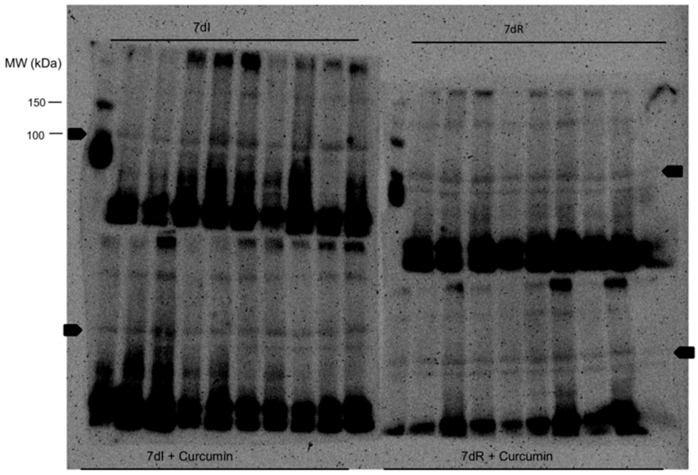

Mañas-García L. et al. Figure S15

NF-κB p50 (50 kDa)

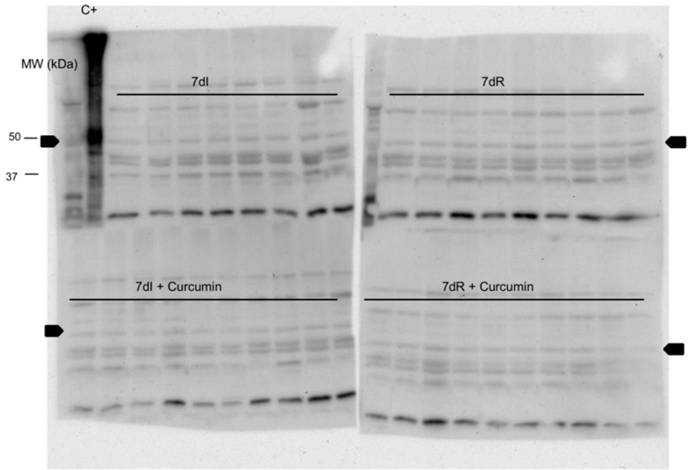

Mañas-García L. et al. Figure S16

Acetylated-NF-κB p50 (50 kDa)

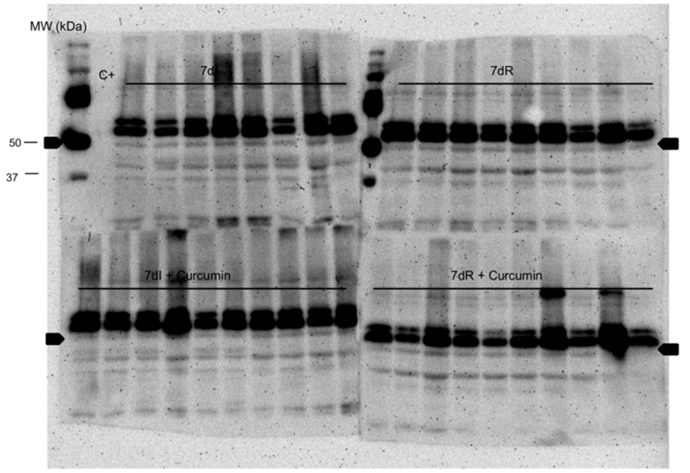

Mañas-García L. et al. Figure S17

FoxO1 (98 kDa)

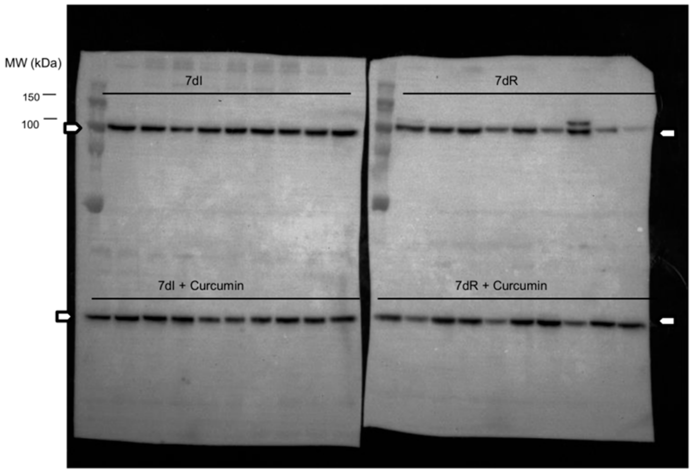

Mañas-García L. et al. Figure S18

Acetylated-FoxO1 (98 kDa)

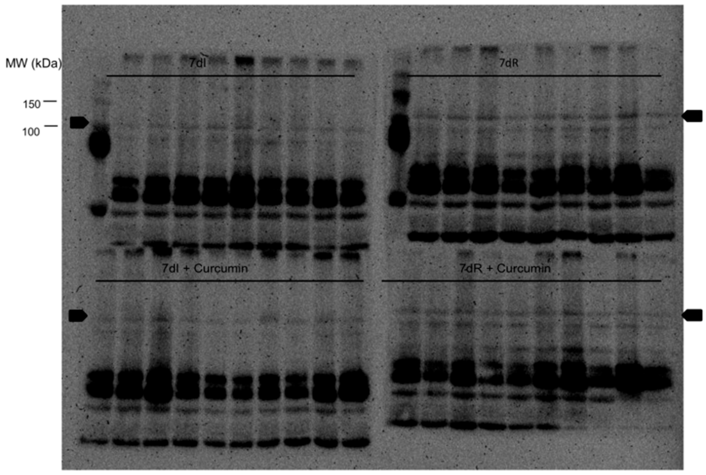

Mañas-García L. et al. Figure S19

Phospho-FoxO1 (98 kDa)

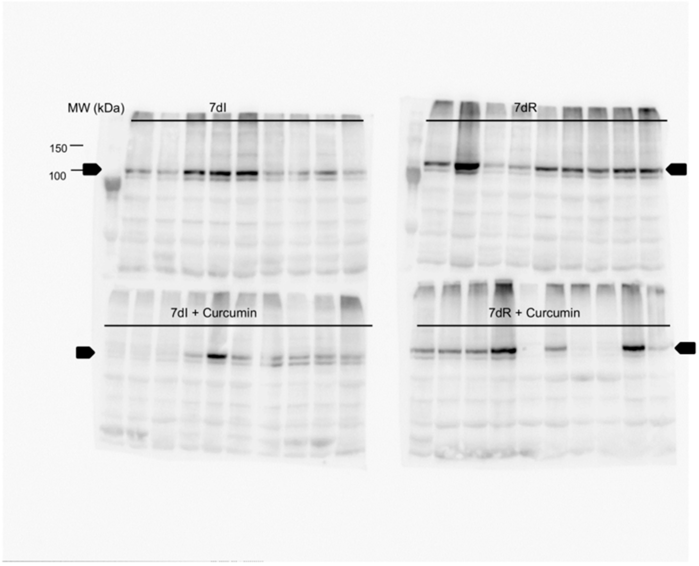

Mañas-García L. et al. Figure S20

FoxO3 (81 kDa)

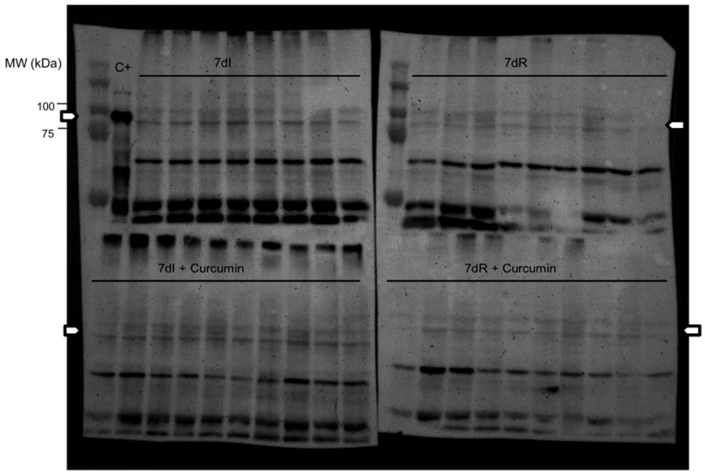

Mañas-García L. et al. Figure S21

Acetylated-FoxO3 (81 kDa)

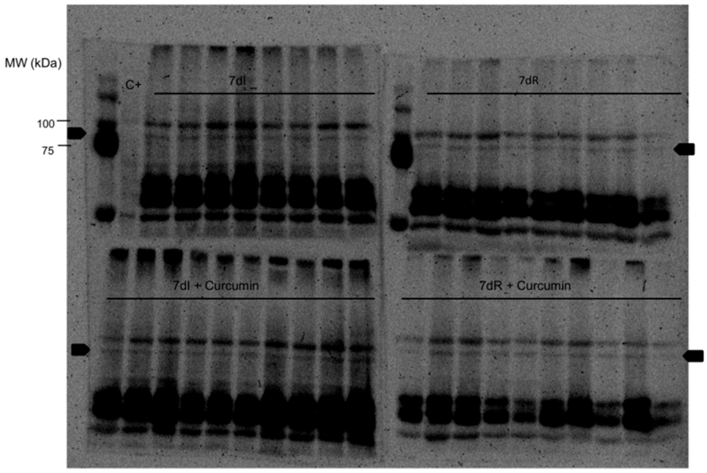

Mañas-García L. et al. Figure S22

Phospho-FoxO3 (81 kDa)

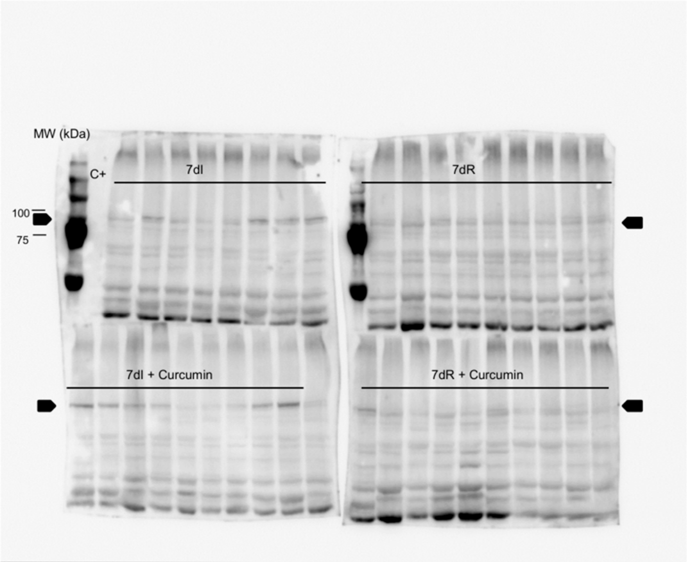

Mañas-García L. et al. Figure S23

HDAC3 (49 kDa)

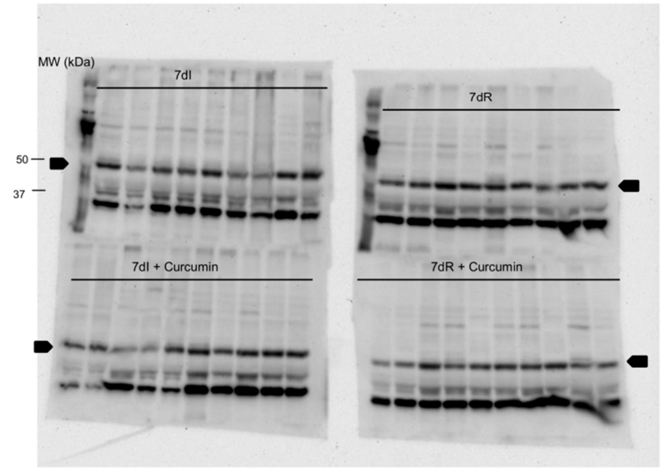

Mañas-García L. et al. Figure S24

HDAC4 (110 kDa)

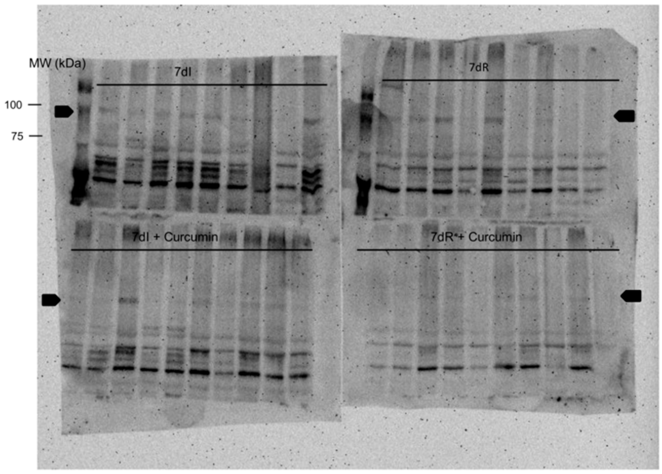

Mañas-García L. et al. Figure S25

HDAC6 (100 kDa)

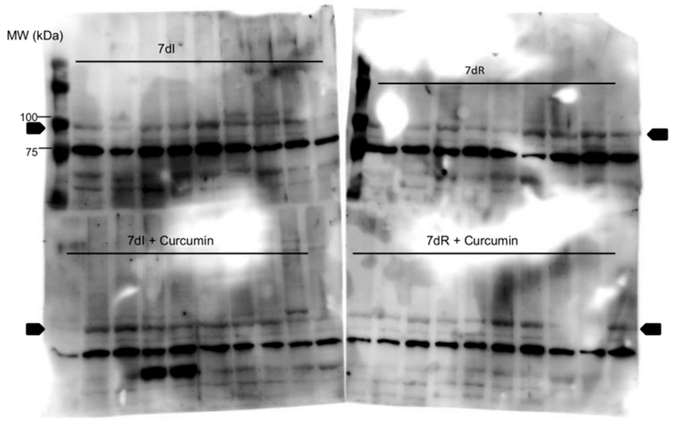

Mañas-García L. et al. Figure S26

GAPDH (37 kDa)

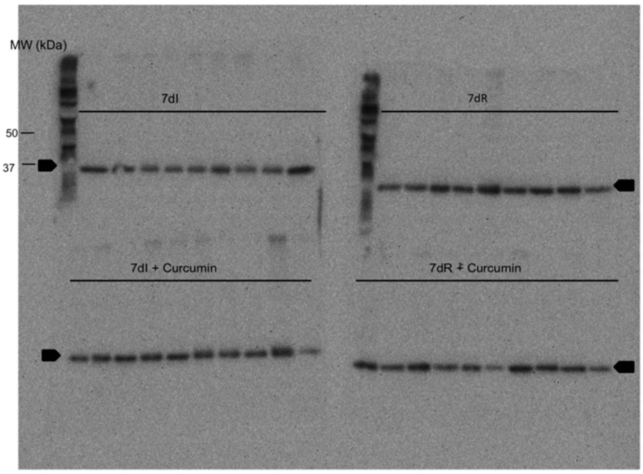

Supplement: Supplementary file 1 [file nutrients-12-00388-s001.pdf]
